# Structural and genetic convergence of HIV-1 neutralizing antibodies in vaccinated non-human primates

**DOI:** 10.1371/journal.ppat.1009624

**Published:** 2021-06-04

**Authors:** Fangping Cai, Wei-Hung Chen, Weimin Wu, Julia A. Jones, Misook Choe, Neelakshi Gohain, Xiaoying Shen, Celia LaBranche, Amanda Eaton, Laura Sutherland, Esther M. Lee, Giovanna E. Hernandez, Nelson R. Wu, Richard Scearce, Michael S. Seaman, M. Anthony Moody, Sampa Santra, Kevin Wiehe, Georgia D. Tomaras, Kshitij Wagh, Bette Korber, Mattia Bonsignori, David C. Montefiori, Barton F. Haynes, Natalia de Val, M. Gordon Joyce, Kevin O. Saunders

**Affiliations:** 1 Duke Human Vaccine Institute, Duke University Medical Center, Durham, North Carolina, United States of America; 2 Department of Medicine, Duke University Medical Center, Durham, North Carolina, United States of America; 3 Emerging Infectious Diseases Branch, Walter Reed Army Institute of Research, Silver Spring, Maryland, United States of America; 4 U.S. Military HIV Research Program, Walter Reed Army Institute of Research, Silver Spring, Maryland, United States of America; 5 Henry M. Jackson Foundation for the Advancement of Military Medicine, Bethesda, Maryland, United States of America; 6 Center for Molecular Microscopy, Center for Cancer Research, National Cancer Institute, National Institutes of Health, Frederick, Maryland, United States of America; 7 Cancer Research Technology Program, Frederick National Laboratory for Cancer Research, Leidos Biomedical Research Inc., Frederick, Maryland, United States of America; 8 Vaccine Research Center, National Institute of Allergy and Infectious Diseases, National Institutes of Health, Bethesda, Maryland, United States of America; 9 Department of Surgery, Duke University Medical Center, Durham, North Carolina, United States of America; 10 Center for Virology and Vaccine Research, Beth Israel Deaconess Medical Center, Boston, Massachusetts, United States of America; 11 Department of Pediatrics, Duke University Medical Center, Durham, North Carolina, United States of America; 12 Department of Molecular Genetics and Microbiology, Duke University Medical Center, Durham, North Carolina, United States of America; 13 Department of Immunology, Duke University Medical Center, Durham, North Carolina, United States of America; 14 Los Alamos National Laboratory, Los Alamos, New Mexico, United States of America; 15 Laboratory of Infectious Diseases, National Institute of Allergy and Infectious Diseases, National Institutes of Health, Bethesda, Maryland, United States of America; The Scripps Research Institute, UNITED STATES

## Abstract

A primary goal of HIV-1 vaccine development is the consistent elicitation of protective, neutralizing antibodies. While highly similar neutralizing antibodies (nAbs) have been isolated from multiple HIV-infected individuals, it is unclear whether vaccination can consistently elicit highly similar nAbs in genetically diverse primates. Here, we show in three outbred rhesus macaques that immunization with Env elicits a genotypically and phenotypically conserved nAb response. From these vaccinated macaques, we isolated four antibody lineages that had commonalities in immunoglobulin variable, diversity, and joining gene segment usage. Atomic-level structures of the antigen binding fragments of the two most similar antibodies showed nearly identical paratopes. The Env binding modes of each of the four vaccine-induced nAbs were distinct from previously known monoclonal HIV-1 neutralizing antibodies, but were nearly identical to each other. The similarities of these antibodies show that the immune system in outbred primates can respond to HIV-1 Env vaccination with a similar structural and genotypic solution for recognizing a particular neutralizing epitope. These results support rational vaccine design for HIV-1 that aims to reproducibly elicit, in genetically diverse primates, nAbs with specific paratope structures capable of binding conserved epitopes.

## Author summary

The primate immune system generates a diverse repertoire of pathogen-binding proteins called antibodies. Primate antibody repertoires must be diverse to respond to infection by many different pathogens. In this study, we characterized the structure and genetic features of HIV-1 inhibitory antibodies from vaccinated monkeys as a model for human vaccination. We found that individual monkeys responded to HIV vaccination by generating highly similar HIV-1 neutralizing antibodies. The antibodies had nearly identical structures and genetic makeups. Thus, the immune systems of different monkeys generate a common solution for inhibiting viral protein function and infectivity. Success of vaccines in multiple recipients may be augmented by the ability of immune systems to reproducibly devise a common antibody solution to attack vulnerable sites on pathogens.

## Introduction

In a recent nonhuman primate vaccine study, vaccine-elicited HIV-1 nAb titers were shown to correlate with protection from the vaccine-matched challenge virus [1]. This correlation, in conjunction with protection by passively-infused antibodies [2,3], provides a rationale for induction of nAbs as a goal for a protective HIV-1 vaccine [4].

HIV-1 nAbs can be categorized based on their neutralization breadth [5,6]. Antibodies that target highly conserved epitopes on HIV-1 envelope glycoprotein are capable of neutralizing diverse HIV-1 isolates and are designated as broadly neutralizing antibodies (bnAbs) [7–9]. These bnAbs are rarely elicited by vaccination in primates [10,11], and in the select cases where they have been induced, they appear at low titers [12,13]. Another category of nAbs are antibodies capable of neutralizing only the HIV-1 strain used for vaccination or infection—also referred to as the autologous virus [1,14–16]. These autologous neutralizing antibodies have been more readily elicited with vaccination than bnAbs.

Autologous neutralizing and broadly neutralizing categories of antibodies are not mutually exclusive. The study of bnAb lineage development and autologous virus coevolution has shown that the early members of bnAb lineages exhibit neutralization activity against autologous HIV-1 isolates only. Upon further affinity maturation, a subset of the bnAb lineage members develop broad neutralization activity [17–19]. Therefore, it is necessary to characterize the epitopes of autologous nAbs to distinguish antibodies in the early stages of bnAb development from those that target a non-conserved epitope.

HIV-1 nAb responses vary in potency, breadth, and epitope specificity during human infection and vaccination; presenting a challenge for HIV-1 vaccines that aim to reproducibly elicit neutralizing antibodies [1,11,20–25]. The variability in antibody responses to envelope may be because the antibody repertoire of an individual is derived from unique rearrangements within each antibody of polymorphic variable, diversity, and joining gene segments [26,27]. Thus, whether uniform HIV-1 nAbs can be consistently induced in multiple primates with vaccination remains a significant question to address. During natural infection, bnAbs with very similar binding modes and similar immunogenetics have developed against the CD4 binding site and membrane proximal external region in multiple individuals [28–33]. Thus, it is possible for the immune system to respond to Env antigen during infection in a reproducible way that results in very similar bnAbs. Two of the most widely used strategies for HIV-1 vaccine design, B cell lineage design and reverse vaccinology design, are built upon the premise that vaccination can also elicit very similar envelope-specific antibodies in multiple individuals [9,34,35]. Each of these strategies aim to elicit the same type of neutralizing antibody in multiple individuals by targeting precursors of specific B cell lineages with HIV-1 envelope immunogens [9,34,35]. In support of these vaccine design concepts, relatively easy-to-induce non-nAbs with conserved binding modes to HIV-1 envelope and similar immunogenetic sequence motifs have been elicited [36]. Determining whether vaccination can elicit more difficult-to-induce stereotyped nAbs in outbred primates would further support B cell lineage and reverse vaccinology design concepts.

In the present study, we isolated and compared four nAbs from three rhesus macaques that were immunized with a group M consensus envelope called CON-S. Although, the macaques were immunized with different CON-S envelope vaccine regimens, the CON-S nAbs had immunogenetic commonalities, and were able to exchange immunoglobulin chains with each other and still maintain envelope reactivity. The atomic-level structures of the unliganded antigen binding fragment (Fab) from two of these antibodies showed nearly identical paratope conformation. Structures of the antibodies in complex with CON-S envelope trimers showed the four CON-S Env-induced antibodies bound to the same epitope on Env with very similar angles of approach. This study demonstrates proof-of-concept that HIV-1 envelope vaccination reproducibly elicits nAbs with nearly identical binding modes to HIV-1 envelope. These results support HIV-1 vaccine design strategies that aim to reproducibly elicit stereotyped nAbs in outbred primates.

## Results

### Vaccine elicitation of nAbs against HIV-1 CON-S

**Fig 1:**
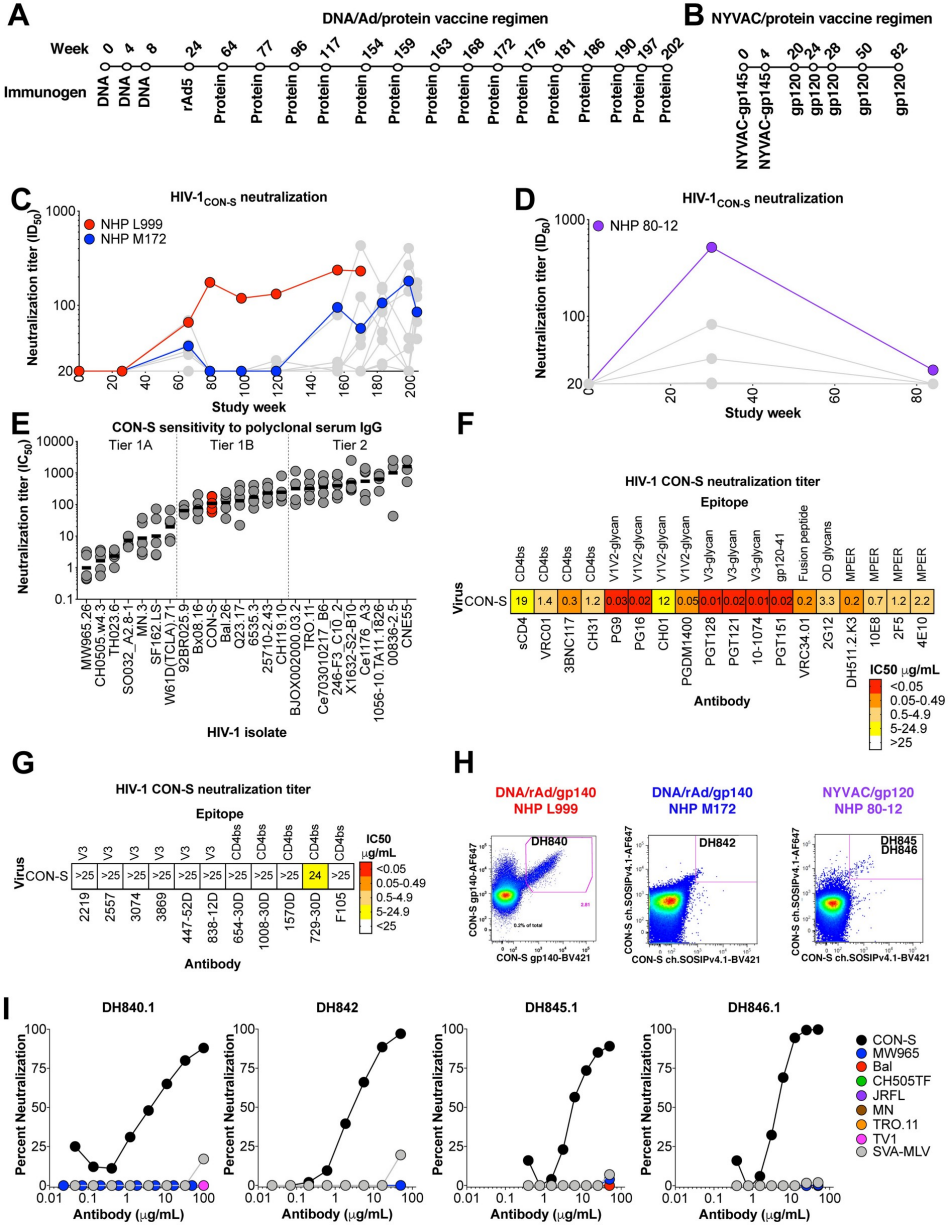
Vaccine elicitation of HIV-1 nAbs in rhesus macaques. **(A)** CON-S envelope DNA/recombinant Adenovirus/protein vaccination regimen administered to macaques L999 and M172 (see Materials and methods for details). (**B)** Macaque 80–12 was immunized with a NYVAC vector expressing gp120 followed by boosting immunization with recombinant gp120. (**C,D)** Macaque serum neutralization of HIV-1 CON-S infection of TZM-bl cells. Serum or plasma was obtained two weeks after immunization and examined for neutralization. Neutralization titer is shown as reciprocal plasma or serum dilution that inhibits 50% of virus replication (ID_50_). Macaques from which monoclonal antibodies were isolated are shown in red, blue, and purple. **(E)** Comparison of neutralization sensitivity of CON-S (red) and other common HIV-1 strains to purified IgG from HIV-1 infected individuals (n = 5). Neutralization titer is shown as IgG concentration in μg/ml that inhibits 50% of virus replication (IC_50_). Horizontal bars represent the geometric mean of the 5 IgG samples. Vertical dotted lines separate different neutralization tiers. **(F,G)** HIV-1 CON-S neutralization sensitivity to **(F)** bnAbs and resistance to **(G)** linear V3 and poorly-neutralizing CD4 binding site antibodies. Neutralization titer is shown as IC_50_, and are color-coded based on potency. **(H)** Antigen-specific single B cell fluorescence-activated cell sorting of the PBMC from each macaque shown in **C** and **D**. The recovered antibody of interest is shown within the magenta sort gate. **(I)** Monoclonal antibody neutralization of HIV-1 infection of TZM-bl cells. Each curve shows the neutralization of different HIV-1 isolates listed in the legend.

Consensus envelopes have been derived from group M HIV-1 isolates as an immunogen design approach for broadening immune responses. In particular, CON-S is central to the M group, and is a consensus of within-clade consensus sequences [37–39]. To investigate nAbs elicited by consensus HIV-1 envelope vaccination, rhesus macaques were immunized with two different vaccine regimens. In one study, Indian origin rhesus macaques were administered CON-S gp140 oligomers via DNA vectors, recombinant Ad5 vectors, and recombinant gp140 protein (Fig 1A) [40]. In the second study, macaques were immunized with NYVAC vectors expressing gp120 and recombinant gp120 protein (Fig 1B) [41]. All of the macaques, including macaques L999 and M172, that received the DNA/rAd5/gp140 vaccine developed nAbs against the autologous HIV-1 CON-S isolate (Fig 1C). Similarly, three of four macaques, including 80–12, that received the NYVAC/gp120 regimen developed nAbs against the vaccine strain (Fig 1D). None of the vaccinated macaques had detectable bnAbs in their plasma or serum (S1 Fig) [41].

Since HIV-1 isolates exhibit a spectrum of neutralizing antibody sensitivities [5,6], we sought to determine the significance of CON-S neutralization by determining its neutralization sensitivity. We determined the neutralization sensitivity tier of CON-S relative to 23 natural HIV-1 isolates [6]. The geometric mean neutralization titer for each virus was determined for purified IgG from 5 HIV-1-infected serum samples. The geometric mean titer for CON-S was 108 μg/mL (Fig 1E), which made it more resistant than the commonly-used natural HIV-1 isolate 92BR025, but more sensitive than routinely-used isolate Q23.17. Overall, CON-S pseudovirus typed as a tier 1B isolate. To determine the exposure of broadly neutralizing and poorly neutralizing epitopes on CON-S envelope, we examined the sensitivity of CON-S pseudovirus to a panel of HIV-1 bnAbs and HIV-1 poorly neutralizing monoclonal antibodies. The bnAbs targeted the CD4 binding site, V1V2-glycan site, V3-glycan, gp120-41 interface, fusion peptide, outer domain glycans, and MPER sites, while the collection of poorly nAbs targeted the third variable region and CD4 binding site. Interestingly, CON-S was sensitive to all bnAbs tested, and was resistant to all poorly neutralizing HIV-1 antibodies (Fig 1F and 1G, two-tailed exact Wilcoxon test, *P*<0.01, n = 11 non-bnAbs versus 17 bnAbs). The difference in sensitivity to broadly versus poorly nAbs could be due to CON-S envelope being most often in a closed conformational state that occludes poorly neutralizing epitopes, or the amino acid sequence of CON-S was sufficiently divergent from each clade resulting in binding by broadly-reactive antibodies only. Nonetheless, the vaccine-elicited macaque serum nAbs targeted epitopes distinct from poorly-neutralizing third variable region-specific or poorly-neutralizing CD4 binding site-specific antibodies.

To determine the epitopes targeted by the vaccine-induced macaque neutralizing antibodies, we sought to isolate monoclonal antibodies from macaques L999, M172, and 80–12—all of which had serum nAbs against CON-S. Single B cells that bound to fluorophore-labeled uncleaved CON-S gp140 were sorted from macaque L999 peripheral blood mononuclear cells (PBMC, S2 Fig). The sorted B cells were cultured *in vitro* and the secreted antibody was tested for CON-S neutralization. Of the 11,639 B cell cultures, only 21 B cell culture supernatants exhibited CON-S neutralization activity (S3 Fig). Antibody DH840.1 was recovered from one culture well that exhibited neutralization (Fig 1H, S3 and S4 Figs). The remaining 20 antibodies either failed to amplify by PCR or lacked neutralization activity once tested as a purified monoclonal antibody (S3 Fig). Similarly, single B cells that bound soluble stabilized CON-S Env trimers were sorted from macaques M172 and 80–12 (Fig 1H). Antibody DH842 was recovered from M172 PBMC, and DH845.1 and DH846.1 were isolated from 80–12 PBMC (Fig 1H, S5 and S6 Figs). Clonal lineages for DH845 and DH846 were inferred by Cloanalyst using single B cell PCR sequences (S7A, S7B, S8 and S9 Figs). The antibodies within the DH845 and DH846 clonal lineages bound to soluble stabilized CON-S Env trimers with similar magnitudes (S7C Fig). Next generation sequencing of peripheral blood B cell heavy chain variable (VH) regions identified four VH regions that were inferred to be clonally related to DH840.1. However, none of the VH regions when paired with the DH840.1 light chain exhibited binding to CON-S Env trimers (S7C Fig). Thus, the natural light chains for these antibodies may be important for binding to CON-S Env trimers. We next determined whether DH840.1, DH842, DH845.1, and DH846.1 could bind to heterologous HIV-1 envelopes from CRF02_AG, clade A, and clade C. DH840.1, DH842, DH845.1, and DH846.1 bound to stabilized CON-S SOSIP gp140 trimers, but lacked binding to BG505, T250-4, CH505 w78.33, CH505 w100.B6, and CH848 transmitted/founder SOSIP trimers (S10A Fig). DH840.1, DH842, DH845.1, and DH846.1 were tested for their neutralization activity against 8 HIV-1 isolates including the autologous CON-S virus. All four monoclonal antibodies neutralized CON-S, but lacked heterologous tier 1 or 2 neutralization (Fig 1I). Additional monoclonal antibodies were also cloned from the same single B cell sorts, however none of these antibodies exhibited any CON-S neutralization activity (S11 Fig). Thus, we only focused on further characterization of the four CON-S neutralizing antibodies.

### Immunogenetic commonalities among CON-S neutralizing antibody lineages

To determine whether the four CON-S nAbs were genetically similar, we inferred the gene segment usage and third complementarity determining region (CDR3) lengths for each antibody. Since the rhesus library of reference gene segments continues to expand and each reference library has different genes included, we used three different reference libraries for gene usage inferences (S7A Fig). For clarity we refer below to the inference from the most recent rhesus Cloanalyst library. The four CON-S nAbs were inferred to originate from the same IGHV4-n gene segment (Table 1 and S7A Fig). The VH of the antibodies were also highly similar to IGHV4-e, but IGHV4-n had the highest identity and fewest gaps in our reference library and was thus selected as the most probable inferred germline gene (S12 Fig). Given the high genetic variability in macaques it is likely that the true germline gene segments of DH840.1 and DH846.1 are allelic variants of this reference gene [42,43]. Of the four antibodies DH840.1 and DH846.1 had the most immunogenetic similarities. Despite being isolated from different macaques vaccinated with different regimens, DH840 and DH846 antibody lineages were both inferred to derive from IGHV4-n and IGKV3-d heavy chain and light chain variable gene segments, respectively (Table 1). Additionally, their heavy and light chain CDR3s were the same lengths (Table 1 and S7A Fig). However, the CDR3 amino acid sequences had low sequence identity (S13 Fig), and the antibodies somatically mutated to encode different amino acids (S8, S9 and S13 Figs). Therefore, antibodies DH840.1 and DH846.1 were derived from similar gene segments, but underwent distinct affinity maturation processes. Thus, the four neutralizing monoclonal antibodies were genetically similar, with one pair of antibodies originating from nearly identical gene segment rearrangements.

**Table 1:** Immunogenetics determined by Cloanalyst rhesus macaque library.

Vaccine	Macaque	Antibody	Macaque VH	Macaque JH	HCDR3 length (aa)	Macaque VL	Macaque JL	HCDR3 length (aa)
DNA/Ad5/Protein	L999	DH840.1	IGHV4-n	IGHJ4	15	IGKV3-d	IGKJ4-1	8
DNA/Ad5/Protein	M172	DH842	IGHV4-n	IGHJ5-1	17	IGLV5-e	IGLJ1	9
NYVAC-gp120	80–12	DH845.1	IGHV4-n	IGHJ1	20	IGKV1-Ab	IGKJ2-1	9
NYVAC-gp120	80–12	DH846.1	IGHV4-n	IGHJ4	15	IGKV3-d	IGKJ1-1	8

### Structural convergence of DH840.1 and DH846.1 variable fragments

**Fig 2:**
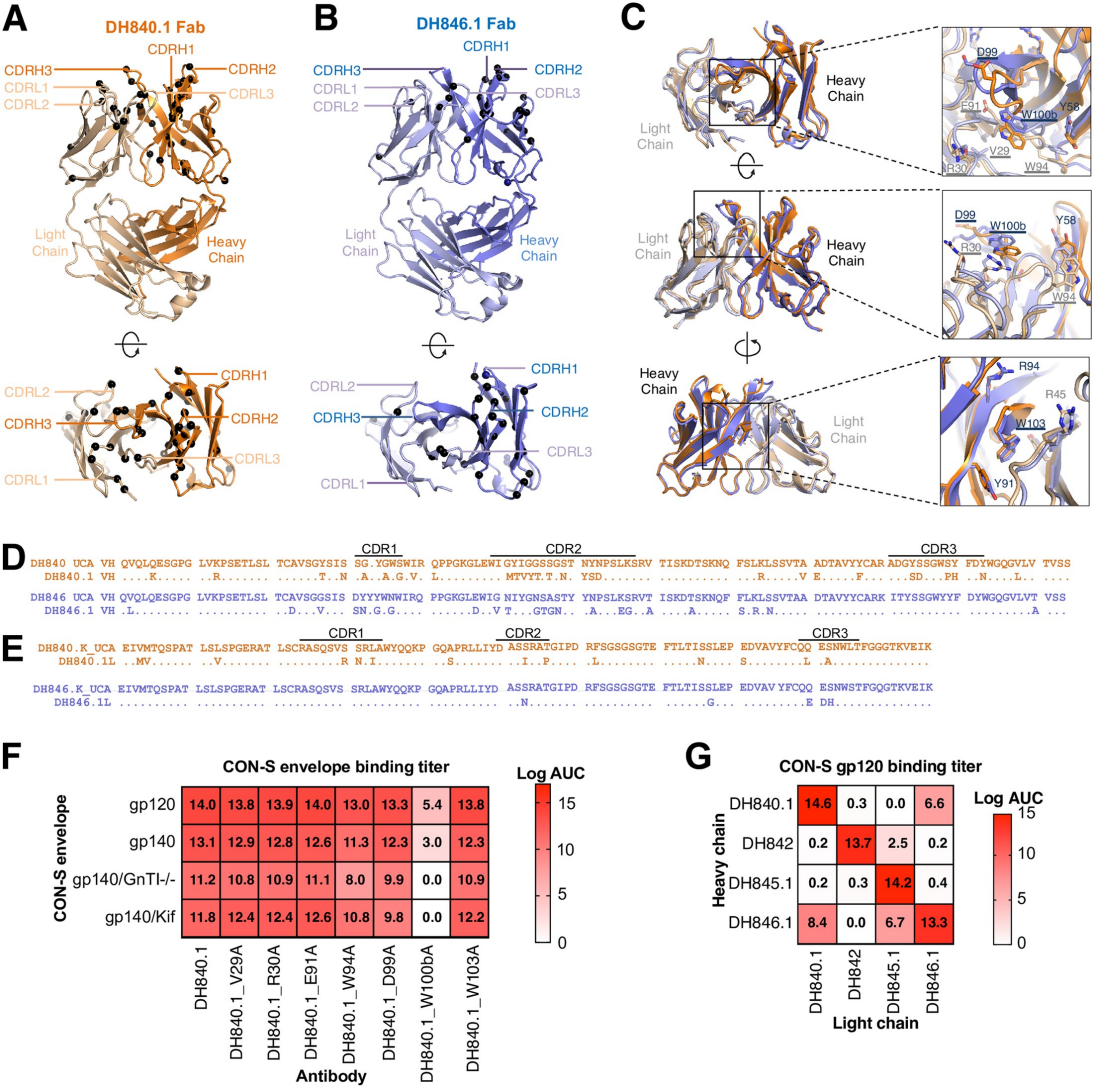
Structural conservation of CON-S neutralizing antibody paratopes. **(A,B)** Crystal structures of **(A)** DH840.1 and **(B)** DH846.1 Fabs shown in ribbon representation in two orientations. Amino acids encoded by nucleotide somatic mutations are indicated by black spheres. **(C)** DH840.1 and DH846.1 Fabs shown in ribbon representation are structurally overlaid and shown in three orientations. Close-up views of select CDR loop residues from both DH840.1 and DH846.1 that are similar in sequence and location are shown in stick representation, with DH840.1 paratope alanine mutants tested in **F** underlined for reference. **(D,E)** Amino acid comparison of DH840.1 (orange), DH846.1 (violet), and their putative unmutated common ancestors. **(D)** Heavy chain and **(E)** light chain variable region amino acid sequences. Dots represent identical amino acids. Variable region domains are listed above the amino acid sequence. **(F)** Antibody binding titers to CON-S envelope by DH840.1 paratope alanine mutants. Binding titer was measured by ELISA, and is shown as the area-under-the-log-transformed curve (log AUC). Kif, kifunensine; GnTI-/-, 293S cells lacking the GnTI enzyme. Mean values of two to four independent measurements are shown. **(G)** Antibody binding titers as log AUC in ELISA to CON-S envelope for chimeric antibodies composed of heavy and light chains from different CON-S nAb lineages. The binding magnitude is color-coded with stronger binding being darker shades of red. Mean values of four independent measurements are shown.

To investigate whether the antibody sequence similarity between DH840.1, and DH846.1 translated to similar variable fragment (Fv) conformation, we determined the crystal structures of unliganded DH840.1 and DH846.1 antigen binding fragments (Fabs) at resolutions of 2.7 Å and 2.8 Å, respectively (Fig 2A and 2B and S1 Table). Superposition of the Fv region of DH840.1 and DH846.1 Fabs showed a high degree of structural overlap, despite the 71.5% heavy chain and 83% light chain sequence identity (Fig 2C and S13 Fig). Within the Fv not only did the peptide backbone overlap, but also the amino acid side chains exhibited the same orientations (Fig 2C). Thus, DH840.1 and DH846.1 were highly similar structurally.

In the DH840.1 and DH846.1 Fab crystal structures, we identified amino acids that were solvent-exposed that could potentially interact with HIV-1 Env. In DH840.1 these amino acids were V29_CDR1_, R30_CDR1_, E91_CDR3_, and W94_CDR3_ in the light chain variable (VL) region (All antibody residue numbering and CDR loops are designated using the Kabat numbering system). The same amino acids were exposed in DH846.1 VL, except a serine was present at position 30_CDR1_ instead of an arginine and aspartate was present at position 91_CDR3_, instead of glutamate (Fig 2D and 2E). In the VH region D99_CDR3_, W100b _CDR3_, and W103 _FR4_ were exposed in the antigen binding site (Fig 2C–2E). W100b _CDR3_ and W103 _FR4_ were oriented almost identically in the DH840.1 and DH846.1 antigen binding fragments (Fig 2C). The DH846.1 VH differed from DH840.1 at position 99_CDR3_ where the inferred germline amino acid serine was encoded (Fig 2C). Among these seven amino acids R30_CDR1_ in the DH840.1 VL, E91_CDR3_ in DH846.1 VL, and D99_CDR3_ in the DH840.1 VH were encoded by somatic mutations (Fig 2D and 2E).

To discern which solvent-exposed amino acids mediated Env binding we mutated each amino acid to alanine and examined binding to HIV-1 envelope. Alanine substitution of W100b_CDR3_ in the heavy chain reduced antibody binding to CON-S by 64% (Fig 2F). The reduction in Env gp120 binding by DH840.1 W100bA was more severe when the envelope glycans were modified to be high mannose glycans by kifunensine treatment or GnT1 knockout (Fig 2F). Changing W94_CDR3_ in the VL also reduced binding activity in the presence of high mannose glycans but to a lesser extent (Fig 2F). Thus, W94_CDR3_ and W100b _CDR3_ enabled DH840.1 to accommodate the presence of high mannose glycans on Env. Although the heavy chain CDR3s of DH840.1 and DH846.1 had low sequence identity, W100b was present in both antibodies (S13 Fig). In summary, one of the principal amino acids that conferred DH840.1 Env binding was a solvent-exposed, germline-encoded tryptophan at position 100b in the heavy chain CDR3.

We hypothesized that the conserved immunogenetics and Fv structures of DH840.1 and DH846.1 would result in antibodies with similar binding modes. Antibodies with highly similar binding modes to Env can exchange immunoglobulin chains and retain binding activity [44,45]. Thus, we exchanged the heavy chain of DH840.1 and DH846.1 with the light chains from each antibody, and assessed CON-S envelope binding. In agreement with the immunogenetic and Fab structure similarities, exchanging heavy chains or light chains between DH840.1 and DH846.1 resulted in chimeric antibodies that still bound to CON-S envelope—albeit weaker than the wildtype antibodies (Fig 2G). We also examined immunoglobulin chain complementation by the other CON-S neutralizing antibodies. The DH845.1 light chain paired with DH846.1 or DH842 heavy chains created less potent, but functional binding antibodies (Fig 2G). DH845.1 heavy chain and DH842 light chain showed limited ability to pair with immunoglobulin chains from the other CON-S neutralizing antibodies. Modeling of the structure of the DH845.1 heavy chain and DH842 light chain suggested the lack of complementation could be due to distinct protruding CDR loops present in each of these two immunoglobulin chains (S14 Fig).

### Glycans at N389, N395, and N460 block CON-S neutralizing antibody binding

The similarity in binding modes among the CON-S nAbs suggested that the antibodies targeted the same epitope on Env. In support of this hypothesis, competition ELISA binding assays showed that all four CON-S nAbs blocked DH840.1 from binding to stabilized CON-S SOSIP gp140 trimer (S10B Fig). To investigate the nature of the epitope recognized by the vaccine-induced CON-S neutralizing antibodies, we assessed their ability to bind to different forms of soluble HIV-1 CON-S envelope. Each of the antibodies bound strongly to gp120 core, gp120, and uncleaved gp140 forms of envelope (S15 and S16 Figs). Thus, antibody binding did not require Env trimers, Env cleavage, or native Env folding (S15 and S16 Figs). When we compared gp120 core binding kinetics, DH840.1 and DH842 exhibited faster off-rates than DH845.1 and DH846.1 (S16B–S16D Fig). Compared to gp120 or uncleaved gp140, binding magnitudes were decreased for stabilized SOSIP gp140 forms of Env (S15A Fig). In particular, DH840.1 and DH842 bound weakest to the CON-S stabilized SOSIP gp140 among the four antibodies. Also, antibody binding was strongest as a bivalent IgG molecule compared to monomeric Fab (S15D Fig). The epitope of the antibodies did not include complex glycans, since binding to Env was unaffected by enrichment of Man_5_GlcNAc_2_, Man_8_GlcNAc_2_, or Man_9_GlcNAc_2_ glycans (S15B and S15E Fig). One exception was the binding of the DH842 Fab, which was inhibited by enrichment of high mannose glycosylation (S15E Fig). We observed stronger binding affinity to deglycosylated gp120 core (S16B–S16D Fig), thus the enrichment for Man_9_GlcNAc_2_ may have introduced a glycoform that sterically hindered binding. Altogether, the results suggested that direct glycan contact was not a principal determinant of antibody binding.

**Fig 3:**
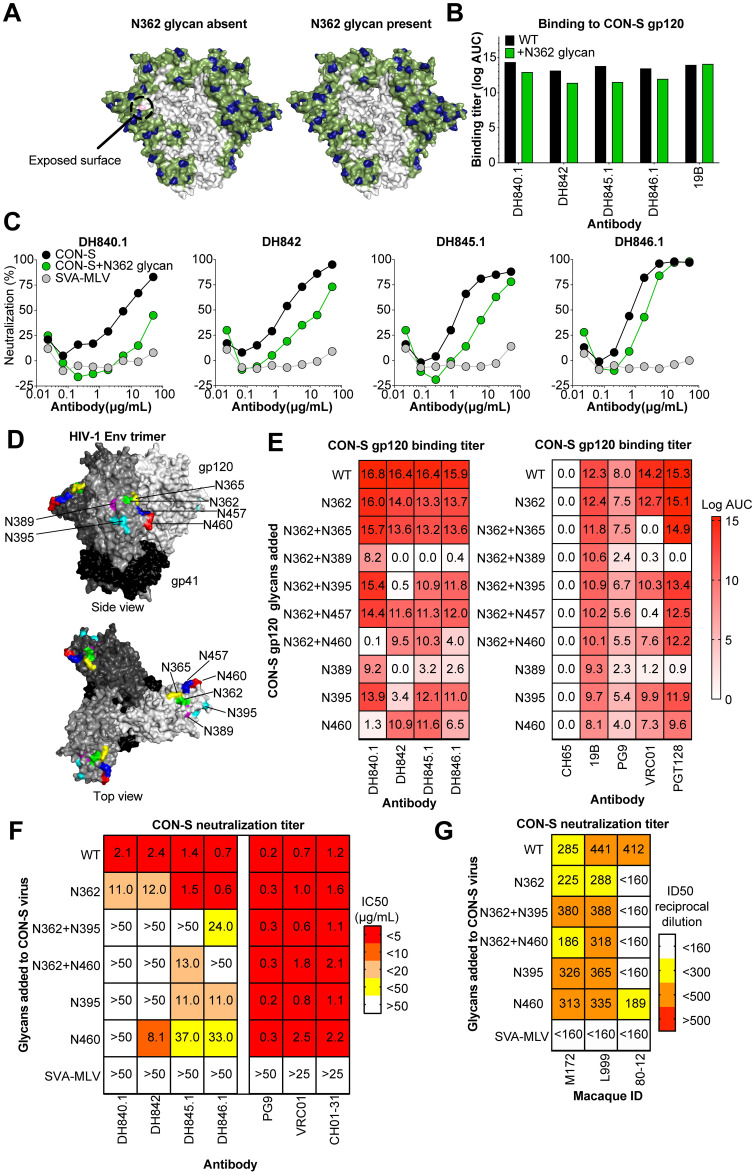
Hyperglycosylation of HIV-1 envelope determines vaccine-induced CON-S nAbs bind the distal side of the HIV-1 envelope gp120 subunit. **(A)** Computational prediction of the CON-S envelope glycan coverage of HIV-1 envelope surface using the Glycan Shield Mapping tool on the Los Alamos HIV Database (https://www.hiv.lanl.gov/content/sequence/GLYSHIELDMAP/glyshieldmap.html). Two protomers of the trimeric envelope is shown in surface representation with potential N-linked glycosylation sites highlighted in blue. The surface potentially covered by a glycan attached to the glycosylation site is shown in green assuming 10 angstrom radius of coverage by each glycan. The gray surface in the center is receding inwards towards the trimer axis, and is predicted to be glycan unshielded; however, it may not be easily accessible to antibodies due to conformational masking from other protomers. (Left) The light pink indicate surface that is covered in 50–80% of group M HIV-1 isolates, but not covered in CON-S. (Right) The addition of a glycosylation site at N362 covers the exposed surface when occupied with glycan. **(B)** Vaccine-elicited neutralizing antibody binding to CON-S gp120 with or without the N362 glycosylation site. Binding titers are shown as log AUC as described in Fig 2. 19B, a V3 region-specific antibody, was used as a positive control. Mean values from two independent experiments are shown. **(C)** Monoclonal antibody neutralization of wildtype (black) and N362 glycan-modified (green) HIV-1 CON-S infection of TZM-bl cells. Murine leukemia virus was used a negative control. Representative results from 2 independent experiments. **(D)** The sites of novel glycan addition in C3, C4, V4, and V5 to block monoclonal antibody binding to CON-S gp120 are shown on the structure of trimeric HIV-1 envelope (PDB:5FYL). Each gp120 of the trimer is colored a different shade of gray and gp41 is colored black. **(E)** Antibody binding titers as log AUC are shown for CON-S gp120 wildtype and hyperglycosylated variants. The glycosylation site added is shown for each row. V1V2-glycan (PG9), CD4 binding site (VRC01), and V3-glycan (PGT128) bnAbs were examined for comparison to vaccine-induced macaque antibodies. 19B is a V3 region-specific antibody. The anti-influenza antibody CH65 was used as a negative control. Mean values of two independent measurements are shown. **(F)** Monoclonal antibody neutralization of infection of TZM-bl cells with wildtype CON-S and CON-S with specified Env glycan additions. Neutralization titers are shown as IC50 in μg/mL and color-coded based on the legend. **(G)** Plasma neutralization of infection of TZM-bl cells with CON-S pseudoviruses shown in **(F)**. Neutralization titers are shown as ID50 reciprocal plasma dilution and color-coded based on the legend.

To determine the Env site of antibody binding, we first mutated known broadly neutralizing epitopes on HIV-1 envelope gp120. Mutation of the V1V2-glycan, V3-glycan, and CD4 binding site had no effect on antibody binding (S15F Fig). Previous studies have shown autologous nAbs can target peptide regions that are not shielded by glycans [46–48]. We predicted the glycan coverage of CON-S to investigate the presence of rare holes in glycan coverage [49]. This analysis showed that the peptide surrounding amino acid 362 was exposed on the CON-S trimer (Fig 3A). This area was predicted to be covered by glycan in 50–80% of HIV-1 isolates (Fig 3A). Of note, the glycan at position 362 in global Env alignments is relatively variable, its frequency is subtype dependent, and the precise location of the glycosylation motif can be shifted by an amino acid or two in different linear sequences while still maintaining the glycan shield in the structure. This variability in the glycosylation site location explains why the structurally conserved glycan was not captured in the single CON-S sequence from 2002, despite glycan shielding in the immediate region being conserved in the structure of most viruses. To determine whether the nAbs targeted this area, we generated CON-S gp140 envelopes with a glycosylation site introduced at N362. The CON-S nAbs bound slightly weaker to CON-S gp140 with the addition of the N362 glycan (Fig 3B). Since recombinant, soluble HIV-1 envelopes can be glycosylated differently than native Env trimers on virions [50–52], we tested neutralization of CON-S with and without the N362 glycan and found between 2 and 10-fold decreases in neutralization potency for three of the four macaque nAbs (Fig 3C). The fourth antibody DH840.1 showed a decrease in maximum percent neutralization from 83% to 45% (Fig 3C). These reductions in neutralization for most of the antibodies, but not complete knockout of activity, suggested the binding site of the antibodies may be in close proximity to amino acid 362. Therefore, we introduced glycosylation sites on the CON-S gp120 to occlude access to sites adjacent to N362 in the envelope tertiary structure. N-linked glycosylation sites were introduced at position 365, 389, 395, 457 and 460 (Fig 3D). The binding of each macaque neutralizing antibody to CON-S gp120 was blocked by one or more of these glycosylation site mutants. N365 and N457 had little effect on antibody binding, but N389, N395, and N460 glycans blocked binding of the antibodies (Fig 3D). The N389 glycosylation site was flanked by N386 and N392 N-linked glycosylation sites, which could affect the glycan processing and occupancy at all three of these sites [53,54]. For some antibodies, the combination of 389, 395, or 460 glycosylation sites with the 362 glycosylation site reduced antibody binding further than the addition of any one glycan site alone. The inhibitory effects of the N389, N395, and N457 glycans were more pronounced when either W94 or W100b amino acids in the DH840.1 paratope were mutated to encode an alanine (S17 Fig). Known V1V2-glycan antibody PG9, CD4 binding site antibody VRC01, and V3-glycan antibody PGT128 showed binding patterns to the CON-S gp120 glycosylation site mutants different from the CON-S nAbs (Fig 3E). This result corroborated the finding that the epitope of the CON-S nAbs was not the V1V2-glycan, V3-glycan, or CD4 binding site (Fig 3E).

We corroborated the effects of adding glycans to envelope regions C3 and V5 on virion-associated Env [50,51]. We generated CON-S pseudoviruses with N-linked glycosylation sites added at positions 365, 389, 395, 457 and 460 alone or in combination with N362 glycan to correspond with the recombinant gp120s described above. Of the 9 CON-S pseudoviruses, only the four viruses with N395, N460, N362/N395, and N362/N460 glycan additions produced infectious virus. Since the glycans were proximal to the CD4 binding site and inhibited VRC01 binding, it is likely that the addition of glycans at 365, 389, and 457 blocked binding to the CD4 binding site rendering the pseudoviruses non-infectious. Nonetheless, the introduction of N362 and N395 glycans eliminated detectable neutralization by three of four antibodies tested (Fig 3F). Similarly, the addition of N362 and N460 glycans eliminated detectable neutralization by three of the antibodies and markedly reduced neutralization by the fourth antibody (Fig 3F). Adding N395 or N460 glycans alone also substantially reduced neutralization activity indicating they were sufficient for inhibiting CON-S autologous neutralizing antibodies. The N395 and N460 glycans did not have an effect on PG9 or VRC01 neutralization of CON-S (Fig 3F). Additionally, N362, N395, and N460 glycans alone or in various combinations inhibited 80–12 plasma neutralization of CON-S (Fig 3G). Thus, the region of Env where C3, V4, and V5 converge were the principal CON-S neutralization determinant for the 80–12 plasma antibody response. L999 and M172 plasma neutralization were not knocked out by these particular glycan additions. Thus, other sites such as 365, 389, and 457 that could not be tested here may have been important for L999 and M172 plasma neutralization. However, the monoclonal antibodies derived from L999 and M172 showed sensitivity to N395, N460, and N362 glycan addition on virion-associated Env trimers indicating antibodies in these macaques targeted the C3-V5 region.

### Structural convergence of HIV-1 neutralizing antibody epitope recognition

**Fig 4:**
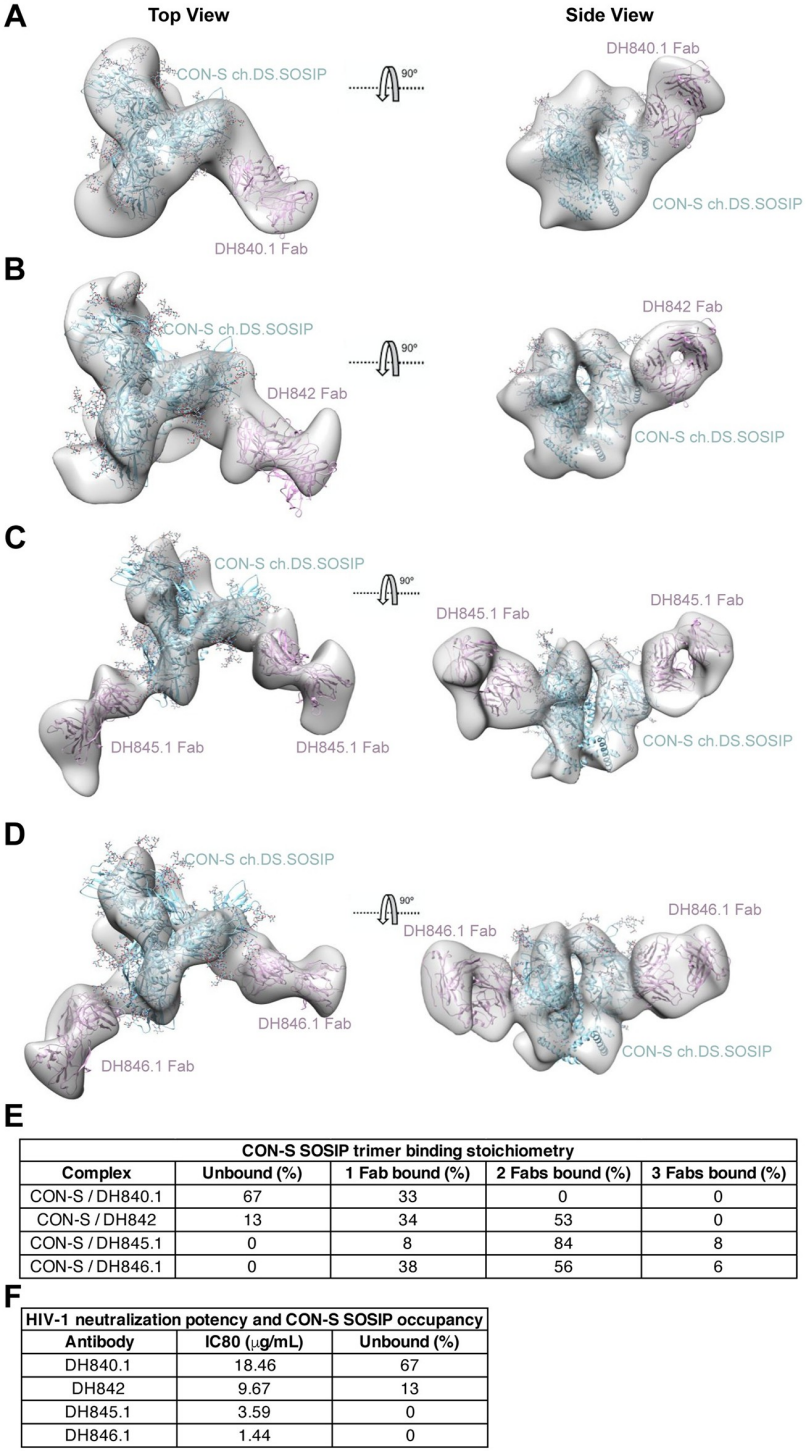
Vaccine-induced CON-S antibodies bind to the outer domain of HIV-1 gp120 near the third constant and fifth variable regions with similar angles of approach. **(A-D)** Top and side views of the final 3D reconstructions of the stabilized soluble CON-S envelope trimer bound to **(A)** DH840.1, **(B)** DH842, **(C)** DH845.1 and **(D)** DH846.1. The 3D volumes are in solid gray with BG505 SOSIP.664 (PDB:4NCO; blue) and Fab (pink) fitted into the density. **(E)** Stoichiometry of Fabs bound to CON-S Env trimer observed by negative stain electron microscopy. **(F)** Comparison of CON-S neutralization potency, shown as the concentration (μg/mL) of antibody that inhibits 80% of virus replication (IC80), and trimer occupancy by at least 1 Fab in negative stain electron microscopy. Stronger neutralizing antibodies exhibited higher stabilized CON-S SOSIP gp140 trimer occupancy in negative stain electron microscopy than weaker neutralizing antibodies.

To definitively show highly similar Env binding modes for each of the CON-S nAbs, we performed negative-stain electron microscopy and 3D reconstruction to map the antigen binding fragment (Fab) of each antibody bound to stabilized soluble CON-S envelope trimer (S18 Fig). The structures of the DH840.1, DH842, DH845.1, and DH846.1 Fab in complex with stabilized soluble CON-S envelope trimer showed each Fab bound to the same region of Env with nearly identical angles of approach (Fig 4). DH840.1 Fab approached the Env tilted more towards the trimer apex than the other three antibodies, but still contacted the same site on Env (Fig 4). The differences in approach angle did not affect antibody binding since the epitope was distal to adjacent protomers of the trimer. The structures were in agreement with the addition of N389, N395, and N460 glycans inhibiting binding, since the attachment sites for these glycans were within the antibody contact region on CON-S envelope trimer (Figs 3D, 3E and 4). Also, DH840.1 bound to third variable (V3) and third constant (C3) region peptides within the identified contact region on a HIV-1 peptide microarray, which was consistent with the contact site shown by the structure (S19 Fig). DH840.1 also bound to a subset of gp41 peptides (S19C Fig). This binding was weaker than binding to gp120 peptides and considered to be non-specific since the antibody-trimer structures showed contact with gp120 and not with gp41. When we examined the glycan shielding of this binding site it was surrounded by glycosylation sites, but several of the sites have been found to be partially occupied on recombinant gp140 proteins (S20 Fig) [53]. Overall, all four antibodies bound to Env gp120 where C3, V3, and fifth variable (V5) regions converged.

Among the four macaque nAbs, there were differences in their binding stoichiometries to CON-S ch.DS.SOSIP. Only 33% of the stabilized soluble CON-S envelope trimer was bound by DH840.1 Fab (Fig 4E). The complexes of DH840.1 Fab and stabilized soluble CON-S envelope trimer showed only a single Fab bound to the trimer (Fig 4E). In contrast, all analyzed stabilized soluble CON-S envelope trimers were bound by at least one DH845.1 or DH846.1 Fab (Fig 4E). The most frequently observed stoichiometry was 2 Fabs per trimer, although 3 Fabs were observed bound to Env trimer occasionally. Three DH842 Fabs were not observed bound to stabilized soluble CON-S envelope trimer (Fig 4E). However, 34% and 53% of analyzed Envs had one or two Fabs bound respectively. Overall, the binding stoichiometries corroborated the binding titers in ELISA, where DH840.1 and DH842 bound weakly, and DH845.1 and DH846.1 bound more strongly to stabilized soluble CON-S envelope trimers (S15A Fig). In total, CON-S nAbs bound to the same region of HIV-1 Env with similar binding modes, but possessed different Fab to Env binding stoichiometries. While a sample size of four antibodies does not comprise a sufficiently large sample size for statistical correlations, we note that the weakest neutralizing antibody, DH840.1, exhibited the highest number of unoccupied trimers and lacked any trimers with 3 Fabs bound (Fig 4F). Both of the two most potent neutralizing antibodies, DH845.1 and DH846.1, had no unoccupied trimers and exhibited 3 Fab to 1 Env trimer binding stoichiometries (Fig 4F). Thus, lower Fab:trimer stoichiometry or occupancy tended to correspond with lower neutralization potency.

### The epitope of the CON-S nAbs is distinct from C3/V4-specific autologous neutralizing antibody CP506

**Fig 5:**
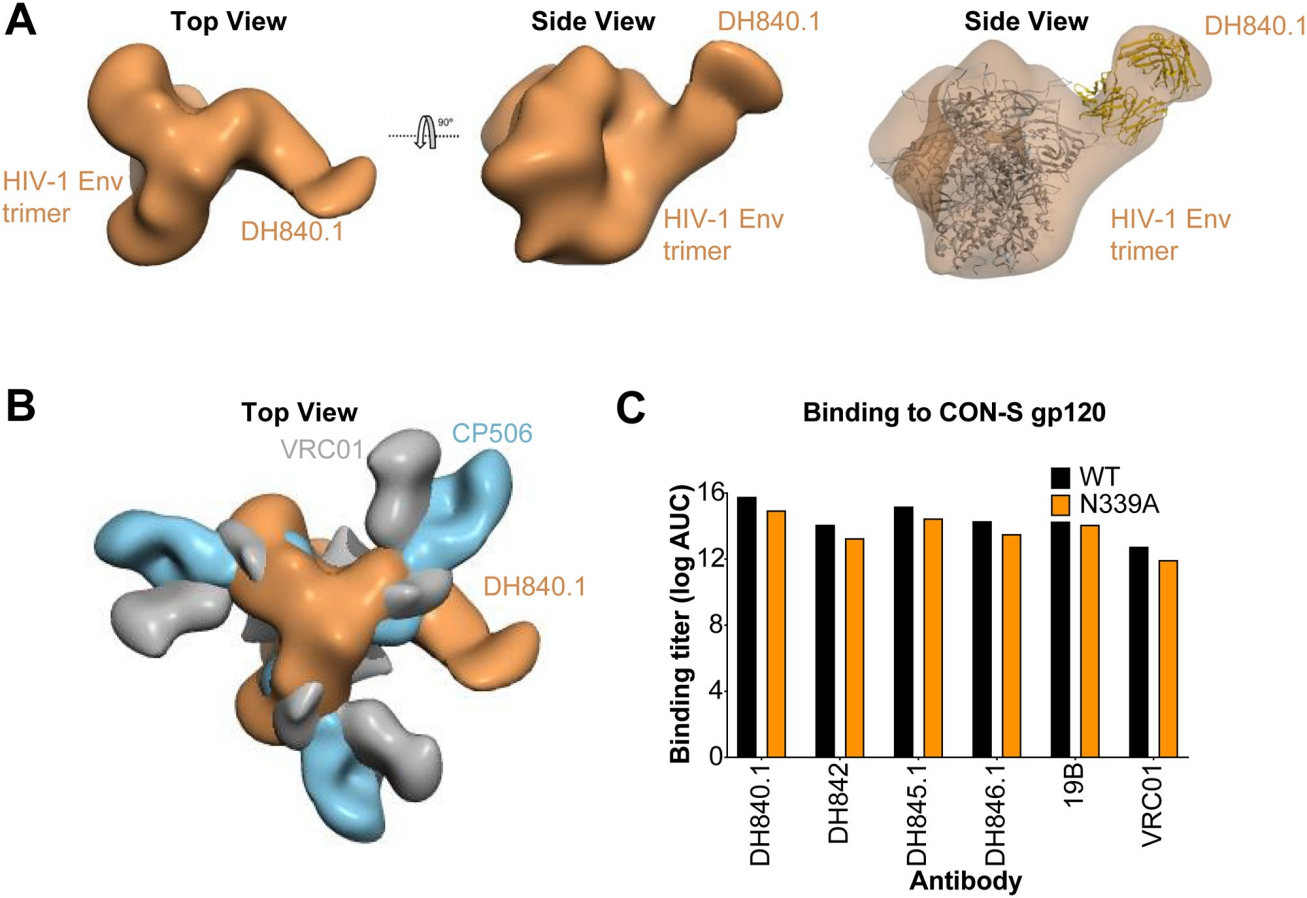
DH840.1 binds to HIV-1 Env in a mode distinct from C3/V4 neutralizing antibodies. **(A)** 3D reconstruction of negative stain electron microscopy of DH840.1 Fab in complex with HIV-1 stabilized soluble CON-S envelope trimer shown from the top or side of the trimer (Left and Middle). Fitting of the HIV-1 Env trimer and DH840.1 Fab into the negative-stain EM map (Right). **(B)** Overlay of the negative-stain EM maps of stabilized soluble HIV-1 CON-S envelope trimer in complex with DH840.1 (colored orange), HIV-1 BG505.SOSIP Env trimer with BG505 autologous neutralizing antibody CP506 (blue; EMDB-9003), and HIV-1 Env with CD4 binding site bnAb VRC01 (gray; EMDB-6193). **(C)** Antibody binding titers as log AUC in ELISA to CON-S gp120 with (black) and without (orange) the N339 glycosylation site.

Next, we sought to determine whether the CON-S nAbs were structurally similar to previously identified monoclonal autologous nAbs. In a previous guinea pig vaccine study, soluble BG505 Env trimers elicited neutralizing antibody CP506 against the C3/V4 region on Env [55]. Additionally, epitope mapping of human serum neutralizing antibody determinants showed serum antibodies targeted the C3/V4 region [56–58]. Given that the CON-S nAbs were blocked by glycans added in the C3 and V4 regions of Env, we compared the structure of antibody CP506 and DH840.1 bound to HIV-1 Env trimer to determine whether they bound to Env in a similar manner. When the 3D-reconstruction of DH840.1 and CP506 were superimposed, it was evident that the angle of approach for each antibody was different (Fig 5A and 5B). While DH840.1 bound to the Env with a perpendicular orientation, CP506 bound in a straight line relative to a given protomer of the trimer (Fig 5B). CP506 was mapped to a novel C3/V4 epitope that included the N339 glycan [55]. The N339 glycan within this region was shown to be required for CP506 binding [55], however elimination of the N339 glycosylation in CON-S gp120 did not knockout DH840.1 binding (Fig 5C). Thus, the angle of approach and dependence on the N339 glycan were different between CP506 and DH840.1 (Fig 5), indicating their epitopes were not the same.

## Discussion

Here, we characterized the immunogenetics, structures, and Env epitopes of neutralizing antibodies induced by HIV-1 vaccination in non-human primates. We identified that non-human primates immunized by different routes with various subunits of the group M consensus envelope CON-S generated genetically similar nAbs to the same epitope on HIV-1 Env. Thus, these antibodies demonstrate clear immunologic conservation, where the primate immune system found a structural, and to a lesser extent immunogenetic, solution for recognition of the same epitope. We term these antibodies the DH840 class of nAbs based on these similarities. A possible explanation for how such antibodies arise in response to vaccination is the presence of common rearranged antibody variable region sequences within the antibody repertoire of different primates. Next generation sequencing of the human antibody repertoire estimates 0.2 to 6% of rearranged antibody variable region sequences are shared between two humans [59,60]. This percentage decreases to 0.02% when comparing antibody repertoires across ten different people [60], but the presence of common rearranged antibody variable region sequences provides the potential for multiple individuals to make genetically and structurally similar antibodies in response to HIV-1 envelope vaccination [61]. Indeed, during human HIV-1 infection, structurally and genetically-convergent nAbs against the CD4 binding site and membrane proximal external region on Env have been isolated [28,32,33]. Similarly, shared HIV-1 antibodies have also been found by next generation sequencing of the antibody repertoire of HIV-infected individuals [61]. With respect to HIV-1 vaccination, non-nAbs that utilized a conserved binding motif to bind the HIV-1 Env second variable region were found in humans and rhesus macaques [36]. Notably, this immunologic phenomena is not specific to HIV-1, as it occurs in response to Malaria or Dengue infection [62,63], as well as *Streptococcus pneumoniae*, influenza haemagglutinin, and Ebola glycoprotein 1 and 2 vaccination [64–66]. Thus, humans have the potential to generate highly similar antibodies to HIV-1 and other pathogens, suggesting the results found in our macaque studies could translate to humans.

Monoclonal antibodies from rabbits and macaques have also shown that regions on Env that lack glycan shielding are a major determinant for autologous neutralizing antibodies [46–48]. Early strain-specific antibody responses in natural infection in humans also tend to focus on such unusual glycan holes [49]. These glycan holes affect the overall nAb response, since bnAb responses develop more rapidly in people infected with viruses that have an intact glycan shield and do not expose strain-specific glycan hole epitopes [49]. The CON-S envelope used here to immunize macaques is predicted to have a nearly complete glycan shield, with only one missing glycan at N362. As noted above, there are two reasons that the relatively conserved glycan shielding around N362 is not represented in the CON-S M group consensus sequence. First, the exact position of the glycosylation motifs among group M HIV-1 isolates shifts relative to HXB2 in a sequence dependent way, and second it is not the most common form in all subtypes. The nearly complete glycan shield of CON-S is analogous to previous glycosylation analyses for another related consensus envelope ConM [67]. Both of these envelopes are group M consensus sequence envelopes, but were inferred from available sequences two years apart. Thus, despite the sequence diversity in group M, deriving a consensus sequence from such a multitude of Envs does not create large glycan-bare holes. This feature of CON-S is in complete agreement with the design expectation and intent; common glycosylation motifs outside of the hypervariable regions are generally readily aligned, with the exception of N355 and N362, and so they are necessarily captured in M group consensus and ancestral immunogens, and a consensus sequence should recapitulate the consensus common glycan shield. While the HIV-1 Env glycan shield remains a difficult challenge for inducing neutralizing antibodies, increasing the completeness of the CON-S glycan shield may further increase the probability of vaccine induction of nAbs that tolerate the full HIV-1 Env glycan shield. Indeed, previous vaccine studies have suggested covering glycan-bare regions on Env can direct the elicited immune response to target desired epitopes [68,69].

The characterization of the DH840 class of antibodies provided new insight into the epitopes targeted by autologous neutralizing antibodies. We mapped the neutralizing epitope to the C3-V5 region, including direct binding to peptides within the V3-C3 junction regions. While this epitope was not a glycan-exposed site, multiple glycosylation sites in this region have low occupancy frequencies on recombinant gp140 proteins [53]. While glycosylation is different on recombinant Env proteins and virion-associated Env [52], underoccupancy of glycosylation sites on virions could facilitate binding to this epitope. In previous vaccine studies, serum antibodies from vaccinated macaques were sensitive to mutations in HIV-1 Env at 332, 160, 356, and 465 [67,70] It is plausible that the antibodies that have mapped to amino acids at 356 and 465 [70] would bind to a very similar epitope. Furthermore, negative stain electron microscopy of polyclonal serum Fabs from BG505 SOSIP gp140-immunized macaques showed that vaccination elicited antibodies against the C3-V5 region of envelope where DH840 class antibodies bound [1]. In a later study, four neutralizing antibodies were isolated from BG505-immunized macaques and shown to bind to the C3-V5 region [71]. The autologous BG505-neutralizing antibodies were derived from a VH4 and VK1 pairing similar to DH845.1. Thus, the similarities between CON-S and BG505 neutralizing antibodies raises the hypothesis that DH840-like antibodies may be a common vaccine-elicited antibody response. The existence of a common antibody class in the macaque germline antibody repertoire capable of binding the C3-V5 region may explain why this antibody response is found in both BG505-immunized and CON-S immunized macaques.

Two of the autologous CON-S neutralizing antibodies exhibited binding stoichiometries less than 3 Fabs per HIV-1 Env trimer. Unoccupied protomers of the Env trimer have been seen in structures of PGT151 and VRC01 bound to HIV-1 Env trimers [72–75]. Although it remains unclear why the CON-S neutralizing antibodies displayed different stoichiometries, possible explanations include antibody-antigen off-rate, conformational changes in unbound protomers, reorientation of the HIV-1 envelope glycan network, or technical artifact due to negative stain electron microscopy buffers and grid preparation [72–75]. Among these potential explanations, antibody-antigen off-rate is supported by our current results. DH840.1 and DH842 exhibited the lowest binding to Env trimer, fastest off-rate, and also the lowest trimer occupancy. The binding stoichiometry of CON-S neutralizing antibodies may be important for neutralization potency, since DH840.1 exhibited the lowest trimer occupancy and the lowest neutralization potency. These results are consistent with previous observations that indicated the proportion of occupied Env trimer directly correlates with increased neutralization [76].

Currently, our structural comparisons suggest autologous neutralization can be conferred by at least three different C3-dependent antibody responses. The first response is a DH840-like antibody response that is focused on the V3-C3 junction and V5 and does not require N339 glycan. The second response is composed of antibodies like CP506, which focus on the C3 and V4 region and require the N339 glycan [55]. A third type of C3-region-dependent antibody response arises during clade C infection, and is inhibited by the presence of a N339 glycan and positive charge at amino acid 350 [56, 57, 58]. Monoclonal antibody CAP88-CH06 is an example of this antibody response [56]. Thus, the effect of the N339 glycan on antibody binding and antibody binding angle distinguish these different antibody responses into 3 distinct categories.

The C3-V5 neutralizing epitope was distinct from the known eight conserved broadly neutralizing epitopes defined by bnAbs from natural infection [9]. It remains to be determined whether antibodies against the C3-V5 epitope can attain neutralization breadth. Despite, there being a small number of relatively conserved amino acids within each of these contact regions, the high degree of sequence variability in these two regions would suggest it would be challenging.

The reproducible elicitation of DH840 class antibodies with vaccination of macaques supports the premise behind B cell lineage design and reverse vaccinology [9,34,35]. These vaccine design strategies aim to elicit in most vaccine recipients the same types of antibodies previously identified in a subset of HIV-infected individuals. Reverse vaccinology identifies an antibody of interest and then designs an immunogen that optimally interacts with that antibody or its precursors in hopes of eliciting such antibodies with vaccination [34]. While this approach relies on structure-based design to optimize the immunogen for binding to the antibody of interest, it also relies on individuals from diverse genotypes generating antibodies that bind in an identical manner as the original antibody of interest. B cell lineage vaccine design has similar requirements for a stereotyped antibody response. This vaccine strategy aims to use a series of immunogens that can stimulate neutralizing antibody precursors and select antibodies undergoing affinity maturation in a manner analogous to the evolution of a known broadly neutralizing antibody [35]. We show here, with vaccine-induced monoclonal antibodies from three different macaques that nAbs can have conserved Env binding modes and genetic characteristics. The antibodies elicited here were strain-specific neutralizing antibodies, and not the bnAbs sought in the reverse vaccinology and B cell lineage design approaches. The next step for a HIV vaccine will be to elicit such phenotypically and genotypically conserved responses against a broadly neutralizing epitope, instead of a strain specific epitope. Such an achievement would allow for consistent engagement of bnAb B cell lineages and subsequent vaccine-induced antibody maturation to produce bnAbs. Thus, vaccine induction of DH840 class antibodies provides a rationale for reverse vaccinology and B cell lineage design approaches that rely on relatively uniform antibodies being elicited by vaccination in diverse individuals.

## Materials and methods

### Animals and immunizations

The rhesus macaque vaccinations were performed as previously described [40,41]. Briefly, Indian origin rhesus macaques were administered intramuscularly CON-S gp140ΔCFI Env DNA (5 mg) at weeks 0, 4, and 8. At week 24, each macaque was immunized with 10^11^ PFU of recombinant adenovirus serotype 5 encoding CON-S gp140ΔCFI Env. Beginning at week 64, the macaques received a series of fifteen 100 μg intramuscular immunizations with CON-S gp140ΔCFI protein formulated with emulsigen and oligo CpG 4–6 weeks apart. Two weeks after the eighth CON-S gp140ΔCFI protein immunization, peripheral blood mononuclear cells (PBMCs) were collected from whole blood for B sorting and cell culture from macaque L999. PBMCs were obtained from macaque M172 two weeks after the thirteenth CON-S gp140ΔCFI protein immunization for B cell sorting. The NYVAC gp120/recombinant gp120 boost consisted of intramuscular immunizations using 10^8^ PFU of NYVAC expressing HIV-1 CON-S gp120 at weeks 0 and 4. At weeks 20, 24, 28, 50, and 82 the macaques were administered intramuscular immunizations with CON-S gp120 protein formulated in a TLR 7, 8, and 9 agonist STR8-C. Whole blood was obtained two weeks after the third gp120 immunization, from which PBMCs were isolated for single B cell sorting. All study procedures were approved by the Duke IACUC and performed at an AAALAC-accredited facility.

### Antigen-specific single B cell sorting

Cryopreserved PBMC were washed, counted, and stained with NK, T, and B cell surface markers and fluorophore-labeled envelope protein for 1 hour at 4 °C [36]. Antibodies used for staining were CD20 FITC clone L27 (BD Biosciences Cat No. 347673), CD3 PerCP Cy5.5 clone SP34-2 (BD Biosciences Cat No. 552852), IgD PE polyclonal (Southern Biotech Cat No. 2030–09), CD8 PE Texas Red clone 3B5 (Invitrogen Cat No. MHCD0817), IgM PE Cy5 clone G20-127 (BD Biosciences Cat No. 551079), CD16 PE Cy7 clone 3G8 (BD Biosciences Cat No. 557744), Live / Dead Aqua (Invitrogen Cat No. L34957), CD14 BV570 clone M5E2 (BioLegend Cat No. 301832), and CD27 APC Cy7 clone O323 (BioLegend Cat No. 302816). Envelope reactive, live, IgD- single B cells were sorted into individual wells of a 96-well PCR plate that contained cell lysis buffer and 5X first-strand synthesis buffer. Plates were frozen on dry ice and ethanol and stored at -80 °C until reverse transcription of RNA. In one case, memory B cells were sorted and cultured at limiting dilution to induce *in vitro* differentiation into antibody-secreting cells using feeder cells and stimulants as previously described [77]. Two weeks later, cultured B cells were transferred into RNALater and culture supernatants were screened for CON-S neutralization. RNA was isolated from B cells that secreted neutralizing antibodies using the RNAeasy kit (Qiagen Cat No. 74181) and stored at -80 °C until used for reverse transcription.

### Rhesus Immunoglobulin RT-PCR

Superscript III (ThermoFisher Cat No. 18080044) and immunoglobulin constant region-specific reverse primers were used to reverse transcribe B cell RNA [36,78,79]. Five microliters of complementary DNA were subjected to two rounds of nested PCR for heavy and light chain variable region amplification. PCR reactions yielding immunoglobulin variable region amplicons were identified by agarose gel electrophoresis. PCR amplicons were purified using the PCR clean-up kit (Qiagen). Purified PCR amplicons were sequenced with 4 μM of forward and reverse primers. Contigs of the amplified immunoglobulin sequences were made, and gene segment usage was inferred with the rhesus library in IMGT V-quest [80] and Clonanalyst [36, 81]. The unmutated common ancestor (UCA) antibodies and antibody phylogenetic trees were inferred using the rhesus library in Cloanalyst [36, 81]. Gene inferences were also determined using the rhesus library in IMGT V-quest [82]. Somatic mutations of the antibodies were determined by aligning nucleotide or amino acid sequences using the program BioEdit (Informer Technologies, Inc.). A second aliquot of the purified PCR amplicon was used for overlapping PCR to generate a linear expression cassette. The expression cassette was transfected into Expi293F cells (ThermoFisher, Cat No. A14527) with Expifectamine (ThermoFisher, Cat No. A14526). Three days after transfection, cell culture media was cleared of cells and secreted recombinant antibodies in the cell culture media were tested for binding to HIV-1 envelope. The variable regions of selected heavy and light chains were synthesized and cloned into gamma, kappa, or lambda expression vectors (GenScript). Plasmids were prepared for transient transfection of Expi293F cells using the MegaPrep plasmid plus kit (Qiagen).

### Recombinant IgG production

Recombinant IgG1 was expressed in Expi293F cells (ThermoFisher, Cat No. A14527) by transient transfection with Expifectamine (ThermoFisher, Cat No. A14526) [11,40]. Five days after transfection cell culture media was cleared of cells by centrifugation and 0.8 μm filtration. IgG1 was purified from cell culture supernatant with protein A (ThermoFisher Cat No. 20334) and antigen binding fragments were purified with KappaSelect or LambdaSelect (GE Healthcare Cat No. 17548201 and 17545801) affinity chromatography. Purified protein was buffer exchanged into PBS with successive rounds of centrifugation, filtration, followed by storage at -80°C.

### Direct ELISA

HIV-1 envelope protein (2 μg mL^-1^) in sodium bicarbonate buffer was incubated in sealed 384-well Nunc immunoassay plates (ThermoFisher, Cat no. 464718) overnight at 4°C [40]. Unbound protein was washed away, and the plates were blocked with SuperBlock for 1 h. Superblock was aspirated and 10 μL of serially diluted antibodies were added to the plate for 90 min. Plates were washed with SuperWash, and binding antibodies were detected with 1:30,000 dilution of horse radish peroxidase-labeled anti-IgG Fc antibody (Southern Biotech, SB108a, Cat no. 4700–05). Horseradish peroxidase was detected with 3,3’,5,5’-Tetramethylbenzidine for 15 min and the reaction was stopped by the addition of 1% HCl. The absorbance at 450 nm of each well was read with a Spectramax plate reader (Molecular Devices). Binding titers were analyzed as area-under-the-curve for log-transformed antibody concentrations.

### Monoclonal antibody competition ELISA

Nunc Maxisorp plates were coated with HIV-1 envelope, washed and blocked with Superblock [40,79]. Non-biotinylated monoclonal antibodies were serially diluted in SuperBlock starting at 100 μg mL^-1^ and incubated with envelope in triplicate wells for 90 min. A subset of wells was left without any antibody competitor. As a negative control, an anti-influenza antibody CH65 was added to the Env prior to addition of the biotinylated monoclonal antibodies. As a positive control, non-biotinylated DH840.1 IgG was used. After 90 min the non-biotinylated antibody was washed away and biotinylated DH840.1 IgG was incubated in the wells for 1 h at a concentration approximating its 50% effective concentration. Each well was washed, and binding of biotinylated antibodies was determined with a 1:30,000 dilution of HRP-conjugated streptavidin. HRP was detected with 3,3’,5,5’-Tetramethylbenzidine and stopped with 1% HCl. The absorbance at 450 nm was read with a Spectramax plate reader (Molecular Devices). Binding of the biotinylated DH840.1 antibody to HIV-1 envelope in the absence of competing antibody was compared to binding in the presence of competing antibody to calculate percent inhibition of binding. Assays were considered valid if the positive control antibodies blocked greater than 20% of the biotinylated antibody binding [40,79].

### HIV-1 Env peptide array

HIV-1 peptide libraries were generated from group M, clade A, clade B, clade C, clade D, CRF01, and CRF02 consensus gp160s [83]. In addition to these gp160s, we also generated peptides from the gp120 of HIV-1 vaccine strains MN, A244, TH023, TV-1, ZM651, and 1086C. Each peptide spanned 15 amino acids and overlapped with neighboring peptides by 12 amino acids. Array slides were obtained from JPT Peptide Technologies GmbH (Germany) by printing a library of peptides onto epoxy glass slides (PolyAn GmbH, Germany). Three identical subarrays were blocked for 1 h, followed by a 2-h incubation with monoclonal antibody, and a subsequent 45-min incubation with anti-monkey IgG conjugated with AF647 (Jackson ImmunoResearch, PA). Array slides were scanned at a wavelength of 635 nm using an InnoScan 710 scanner (InnopSys, Denmark) and images were analyzed using Magpix V8.1.1 and visualized with R statistical package (R Foundation for Statistical Computing).

### HIV-1 sequence analysis

The prevalence of amino acids at each position between 326–346 was determined using AnalyzeAlign (https://www.hiv.lanl.gov/content/sequence/ANALYZEALIGN/analyze_align.html). LOGO plots were generated by AnalyzeAlign.

### Recombinant HIV-1 gp140 SOSIP production

Soluble HIV-1 Env trimers were expressed, purified and characterized as previously described [11,84]. The soluble CON-S envelope trimer was designed as a stabilized CON-S SOSIP gp140 and was expressed in Freestyle293 cells (ThermoFisher Cat No. K900001) by transient transfection with plasmid DNA complexed with 293Fectin (ThermoFisher Cat No. 12347019). 650 μg of SOSIP expressing plasmid and 150 μg of furin expressing plasmid were used for each 1L of cells. Cell-free culture supernatant was 0.8 μm filtered and concentrated to approximately 100 mL. The SOSIP protein was purified from the cell culture supernatant with PGT145 affinity chromatography. Cell-free supernatant was applied to the column at 2 mL min^-1^ using an AKTA Pure (GE Healthcare Cat No. 29018224), washed, and protein was eluted off the column with 3M MgCl_2_. The eluate was immediately diluted in 10 mM Tris pH 8, 0.2 μm filtered, and concentrated down to 2 mL for size exclusion chromatography. Trimeric protein was purified with a Superose6 16/600 column (GE Healthcare Cat No. 29323952) in 10 mM Tris pH 8, 500 mM NaCl. Trimeric HIV-1 Env protein was sterile-filtered, snap frozen, and stored at -80°C. The formation of trimers was determined by negative stain electron microscopy, blue native-PAGE, and analytical size exclusion chromatography.

### Recombinant gp120, gp140ΔCFI, and gp120 core production

Each HIV-1 envelope construct was expressed by transient transfection using Freestyle 293F (ThermoFisher Cat No. K900001) cells and 293Fectin (ThermoFisher Cat No. 12347019). To modify envelope glycosylation, cells were treated with 5 μM kifunensine. Additionally, envelope was expressed in 293S GnT1^-/-^ cells (ATCC, Cat No. CRL-3022) to restrict glycan processing. HIV-1 gp120 and gp140ΔCFI were purified by lectin chromatography followed by Superdex200 (GE Healthcare Cat No. 28989335) or Superose6 (GE Healthcare Cat No. 29323952) size exclusion chromatography respectively. The CON-S extended core (core_e_) gp120 was designed as described by Kwong and colleagues [85]. Briefly, a gp120 molecule was constructed using Env residues 44–492 with deleted V1, V2, and V3 loops that were replaced by glycine/serine-linkers. The CON-S gp120 core was purified by nickel chromatography. The HIV-1 CON-S gp120 core_e_ was deglycosylated using endoglycosidase H, and purified with Con A-Sepharose (Millipore Sigma Cat No. GE17-0440-01) negative-selection, followed by Superdex200 (GE Healthcare Cat No. 28989335) size-exclusion chromatography. CON-S core_e_ 3c-His8 sequence (signal peptide is underlined): MDSKGSSQKGSRLLLLLVVSNLLLPQGVVGQVWKEANTTLFCASDAKAYDTEVHNVWATHACVPTDPNPQEIVLENVTENFNMWKNNMVEQMHEDIISLWDQSLKPCVKLTggSAITQACPKVSFEPIPIHYCAPAGFAILKCNDKKFNGTGPCKNVSTVQCTHGIKPVVSTQLLLNGSLAEEEIIIRSENITNNAKTIIVQLNESVEINCTRPNNggsgsgGDIRQAHCNISGTKWNKTLQQVAKKLREHFNNKTIIFKPSSGGDLEITTHSFNCRGEFFYCNTSGLFNSTWIGNGTKNNNNTNDTITLPCRIKQIINMWQGVGQAMYAPPIEGKITCKSNITGLLLTRDGGNNNTNETEIFRPGGGDMRDNWRSELYKYKVVKIEGSLEVLFQGPGHHHHHHHH**

### Site-directed mutagenesis

Single mutations were introduced into the DH840.1 variable regions or CON-S gp120 using the QuikChange Lightning Multi Site-Directed Mutagenesis Kit (Agilent). Oligonucleotides were synthesized and purified by standard desalting (Integrated DNA Technologies). Fifty nanograms of purified DNA was used as the template for mutagenesis. Mutagenesis reactions were conducted per the manufacturer’s instructions except the volume of DpnI was doubled and the digestion was conducted for 1 h.

### *In vitro* HIV-1 neutralization

Antibody-mediated HIV-1 neutralization was measured using Tat-regulated luciferase (Luc) reporter gene expression in TZM-bl cells as described previously [86]. TZM-bl cells were obtained from the NIH AIDS Research and Reference Reagent Program, as contributed by John Kappes and Xiaoyun Wu. Pseudoviruses were produced by transient transfection of 293T cells. [87,88]. The monoclonal antibody was pre-incubated with virus (~150,000 relative light unit equivalents) for 1 h at 37 °C, and TZM-bl cells were subsequently added. After 48 h, cells were lysed and Luc activity determined with BriteLite Plus Reagent (Perkin Elmer) and a microtiter plate luminometer. Neutralization titers are the inhibitory concentration at which relative luminescence units (RLU) were reduced by 50% compared to RLU in virus only control wells after subtraction of background RLU in uninfected cell only control wells (IC50).

### Octet biolayer interferometry

Affinity assays were performed on an Octet RED96 instrument at 30°C with kinetics buffer used for all dilution, baseline and dissociation steps. Anti-Penta-HIS (HIS1K) biosensors (Pall ForteBio), loaded with CON-S core_e_ or Deglycosylated CON-S core_e_ at ~50% of maximum binding capacity (30 μg/ml for 100 s), were dipped into wells containing serial dilutions of the DH840 class antibodies ranging from 2.8 μM to 115 nM for 30–60 s. Antibody-core_e_ complexes were then allowed to dissociate for 30–90 s in kinetics buffer. After reference subtraction, binding kinetic constants were determined, from at least 4 concentrations of DH840 class antibodies, by fitting the curves to a 1: 1 Langmuir binding model using the Octet Data Analysis software v10.0 (Pall ForteBio).

### X-ray crystallography

Purified DH840.1 Fab and DH846 Fab were concentrated to 10 mg/ml and used for crystallization screening. A set of 1200 crystal growth conditions prepared using an Art Robbins Scorpion robot, were assessed by mixing 0.2 μL of protein with 0.2 μl of reservoir solution using the sitting-drop vapor diffusion method at 20°C. Once initial crystal conditions were observed, further crystallization trials to improve crystal size and shape were carried out by hand, using a 1:1 ratio of protein and reservoir solution. DH840.1 Fab crystals were obtained with a reservoir solution containing 20% PEG 8000, 0.2M sodium chloride, 0.1M phosphate-citrate (pH 4.2), and DH846 Fab crystals were obtained with a reservoir solution containing 12.5% MPD, 12.5% PEG1000, 12.5% PEG3350, 0.06M Magnesium chloride hexahydrate, 0.06M calcium chloride dehydrate, 0.1M imidazole-MES pH 6.5. Optimized crystals were briefly soaked in mother liquor supplemented with 20% glycerol for DH840.1 Fab, and with 20% ethylene glycol for DH846 Fab, and cryo-cooled in liquid nitrogen prior to x-ray diffraction data collection.

X-ray diffraction data for DH840.1 Fab crystals were collected at 0.98 Å wavelength at APS, ANL (Advanced Photon Source, Argonne National Laboratory) beamline 22-BM, and data for DH846 Fab crystals were collected at 0.98 Å wavelength at beamline 19-BM. Data collection and refinement statistics are provided in S1 Table. All diffraction data were processed with the HKL2000 suite [89]. Structures were solved by molecular replacement using PHASER with the heavy chain of 6C6X and light chain of 5I16 PDBs as search models for DH840.1 Fab, while the refined structure of DH840.1 Fab was used as a search model for DH846 Fab. Iterative model building and refinement were performed in COOT [90] and Phenix [91], respectively. Prior to refinement, a cross validation (Rfree) test set consisting of 5% of the reflections was selected and used to assess the model accuracy throughout the refinement process. The Ramachandran plot as determined by MOLPROBITY [92] showed 95% of all residues in favored regions and 99% of all residues in allowed regions. Crystal structures were deposited in the Protein Database under identification numbers 6U6M and 6U6O. Structure figures were prepared using PyMOL (The PyMOL Molecular Graphics System (DeLano Scientific).

### Negative stain electron microscopy (NSEM)

The purified HIV chimeric CON-S DS.SOSIP-664 trimers were incubated with DH840, DH842, DH845 and DH846 Fab using a 10-fold molar excess of the Fab. These 4 mixtures were incubated for 1h at room temperature and were analyzed by NSEM. A 3 μL aliquot containing ~0.01 mg/mL of the sample was applied for 20 s onto a carbon-coated 200 Cu mesh grid (Electron Microscopy Sciences, Protochips, Inc.) that had been glow discharged at 30 mA for 30 s (Pelco easiGlow, Ted Pella, Inc.), then negatively stained with 0.7% (w/v) uranyl formate for 40 s. Data for the 4 complexes was collected using a Tecnai FEI T20 electron microscope operating at 200 kV, with an electron dose of ~40 e^-^/Å^2^ and a magnification of 100,000 x that resulted in a pixel size of 2.19 Å at the specimen plane. Images were acquired with an Eagle 2kx2k CCD camera (FEI) using a nominal defocus of 1100 nm and the SerialEM software [93]. For electron microscopy data processing, particles were selected from the micrographs, extracted, and a reference-free 2D class averages were obtained using RELION 2.1.0 [94]. After 2D sorting, particles were subject to 3D classification, requesting 4 classes, and starting with an initial model of the trimer unliganded and filtered to 60 Å resolution without imposing symmetry. In case of the trimer bound to DH840 and DH842, all 4 classes showed only 1 Fab bound to the trimer. For the complexes with DH845 and DH846, all 4 classes showed 2 Fabs bound to the trimer. The best class for every complex was selected for final refinement without imposing symmetry in RELION. The resolutions for the 3D final reconstructions were 29 Å for the trimer bound to DH840.1 (EMDB: EMD-21448), 17 Å for the trimer bound to DH842 (EMDB: EMD-21449), 21 Å for the trimer bound to DH845.1 (EMDB: EMD-21450) and 20 Å for the trimer bound to DH846.1 (EMDB: EMD-21451).

### Statistical analyses

Descriptive statistics were calculated using Prism version 8 (GraphPad). Prism version 8 (GraphPad) was used to perform group comparisons with Exact Wilcoxon tests. Peptide array data were visualized with R statistical package (The R foundation).

## Supporting information

S1 Table Crystallographic data collection and refinement statistics. (PDF)

S1 Fig Plasma antibody neutralization of HIV-1 pseudovirus infection of TZM-bl cells. (PDF)

S2 Fig Fluorescence-activated single-cell sorting of envelope-specific B cells. (PDF)

S3 Fig Small-scale high-throughput characterization of binding reactivity and CON-S neutralization by cell culture supernatants containing monoclonal antibodies from antigen-specific B cells from immunized macaques. (PDF)

S4 Fig Immunogenetic analysis of monoclonal antibody sequences isolated from macaque L999. (PDF)

S5 Fig Immunogenetic analysis of monoclonal antibody sequences isolated from macaque M172 post vaccination. (PDF)

S6 Fig Immunogenetic analysis of monoclonal antibody sequences isolated from macaque 80–12 post vaccination. (PDF)

S7 Fig Clonal analysis of rhesus macaque CON-S neutralizing antibodies. (PDF)

S8 Fig Amino acid alignments of heavy chain variable regions for each CON-S autologous neutralizing antibody clonal lineage. (PDF)

S9 Fig Amino acid alignments of light chain variable regions for each CON-S autologous neutralizing antibody clonal lineages. (PDF)

S10 Fig CON-S autologous neutralizing antibodies lack Env trimer binding breadth. (PDF)

S11 Fig HIV-1 neutralization titers for rhesus monoclonal antibodies induced by CON-S vaccination. (PDF)

S12 Fig Comparison of DH846.1 and DH846.2 nucleotide sequence with putative rhesus macaque germline V gene segments. (PDF)

S13 Fig Amino acid alignment of DH840.1 and DH846.1 variable regions. (PDF)

S14 Fig Model of DH842 and DH845 Fab molecules. (PDF)

S15 Fig Antibody recognition of different CON-S envelope subunits and glycoforms. (PDF)

S16 Fig Binding kinetics of CON-S nAbs to glycosylated and deglycosylated CON-S core_e_ measured by biolayer interferometry. (PDF)

S17 Fig The structure and function of the DH840.1 paratope demonstrates W94CDR3 and W100bCDR3 are critical for DH840.1 binding to hyperglycosylated CON-S envelope gp120. (PDF)

S18 Fig Negative-stain electron microscopy single-particle 2D class averages of HIV-1 CON-S DS.SOSIP.664 trimer bound to (A) DH840.1, (B) DH842, (C) DH845.1, and (D) DH846.1 Fabs. (PDF)

S19 Fig DH840.1 reactivity with HIV-1 envelope gp120 and gp41 peptides. (PDF)

S20 Fig Complete and partially unoccupied glycosylation sites in CON-S gp140. (PDF)
