# Supplementary material for: Structural and genetic convergence of HIV-1 neutralizing antibodies in vaccinated non-human primates
Source: PLoS Pathog. 2021 Jun 4;17(6):e1009624. doi: 10.1371/journal.ppat.1009624 (PMC8216552; doi:10.1371/journal.ppat.1009624)
Supplement: S1 Table — (PDF) [file ppat.1009624.s001.pdf]

S1 Table. Crystallographic Data Collection and Refinement Statistics

	DH840.1 Fab	DH846.1 Fab
PDB ID	6U6M	6U6O
**Data collection**		
Space group	$P2_12_12_1$	$P2_1$
Cell dimensions		
a,b,c (Å)	66.1, 75.1, 93.1	71.0, 37.0, 87.9
α,β,γ (°)	90.0, 90.0, 90.0	90, 1, 112.0, 90
Resolution (Å)	50.0-2.7	50.0-2.8
R_{sym}	20.0 (78.0)	22.8 (75.7)
I / σI	12.0 (3.6)	5.23 (1.6)
Reflections (tot/uni)	55,076/12,547	25,135/9,288
Completeness (%)	93.0 (87.98)	84.5 (81.5)
Redundancy	4.4 (3.6)	2.7 (2.6)
CC(1/2)	0.99 (0.80)	0.95 (0.60)
R_{pim}	8.4 (36.8)	13.0 (43.7)
**Refinement**		
Resolution (Å)	50.0-2.7	50.0-2.8
No. reflections	12,545	9,070
R_{work} / R_{free} *	21.9/28.6	21.3/27.01
Ramachandran favored/allowed/outliers	96.0/3.8/0.2	94.8/5.2/0
B-Factor Protein/ion/water	37.9/-/34.8	50.6/-/37.2
R.m.s deviations Bond lengths (Å)	0.002	0.003
Bond angles (°)	0.57	0.66

Values in parentheses are for highest-resolution shells.

* R_{free} was calculated using ~5% randomly selected reflections.
