# Supplementary material for: Structural and genetic convergence of HIV-1 neutralizing antibodies in vaccinated non-human primates
Source: PLoS Pathog. 2021 Jun 4;17(6):e1009624. doi: 10.1371/journal.ppat.1009624 (PMC8216552; doi:10.1371/journal.ppat.1009624)
Supplement: S1 Fig — (PDF) [file ppat.1009624.s002.pdf]

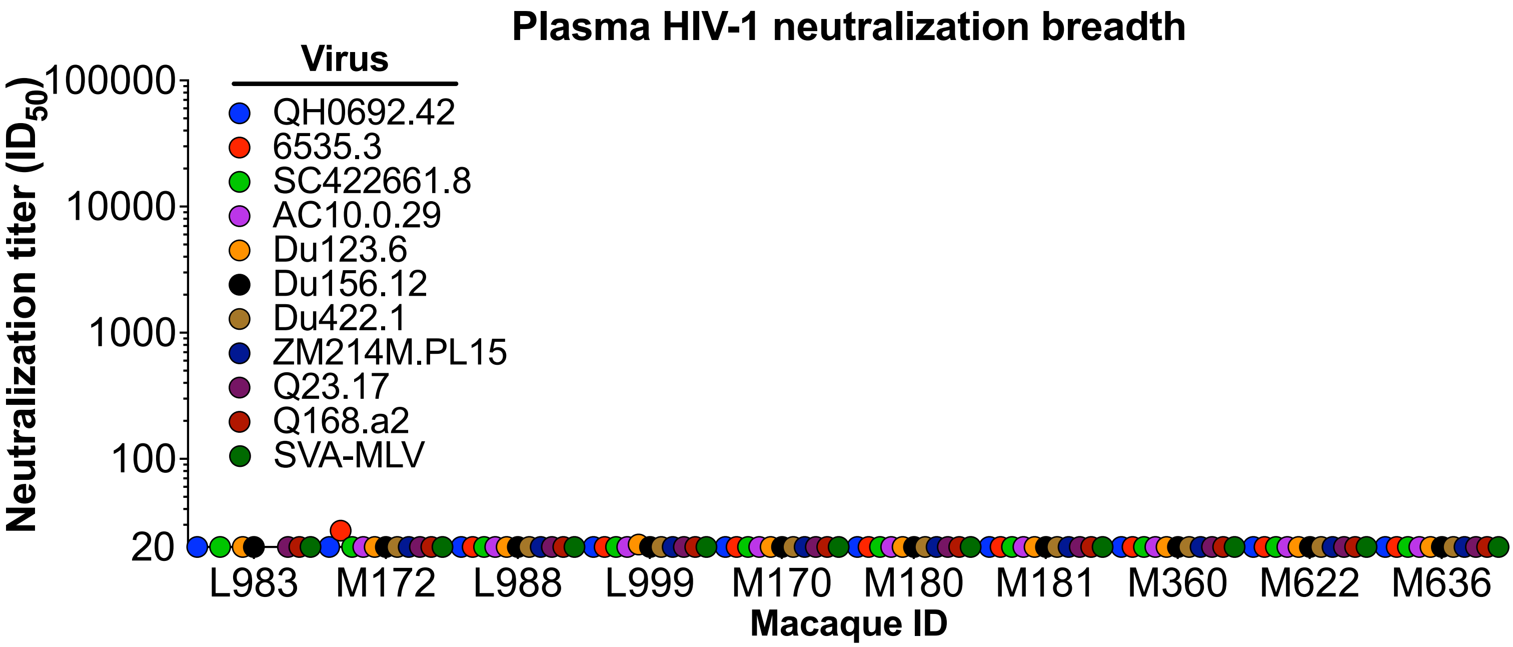

S1 Fig. Plasma antibody neutralization of HIV-1 pseudovirus infection of TZM-bl cells. Plasma obtained two weeks post the final protein boost (week 204) was examined for all macaques except L999. Plasma collected two weeks after the eighth protein boost (week 170) was examined for L999 since it died before the ninth protein immunization. Neutralization titer is shown as the reciprocal dilution of plasma that inhibited 50% of virus replication. The neutralization tiers of the viruses are as follows: Tier 1B - 6535.3, Q23.17; Tier 2 - QH0692.42, SC422661.8, AC10.0.29, Du123.6, Du156.12, Du422.1, ZM214M.PL15, Q168.a2.
