# Supplementary material for: Structural and genetic convergence of HIV-1 neutralizing antibodies in vaccinated non-human primates
Source: PLoS Pathog. 2021 Jun 4;17(6):e1009624. doi: 10.1371/journal.ppat.1009624 (PMC8216552; doi:10.1371/journal.ppat.1009624)
Supplement: S2 Fig — (PDF) [file ppat.1009624.s003.pdf]

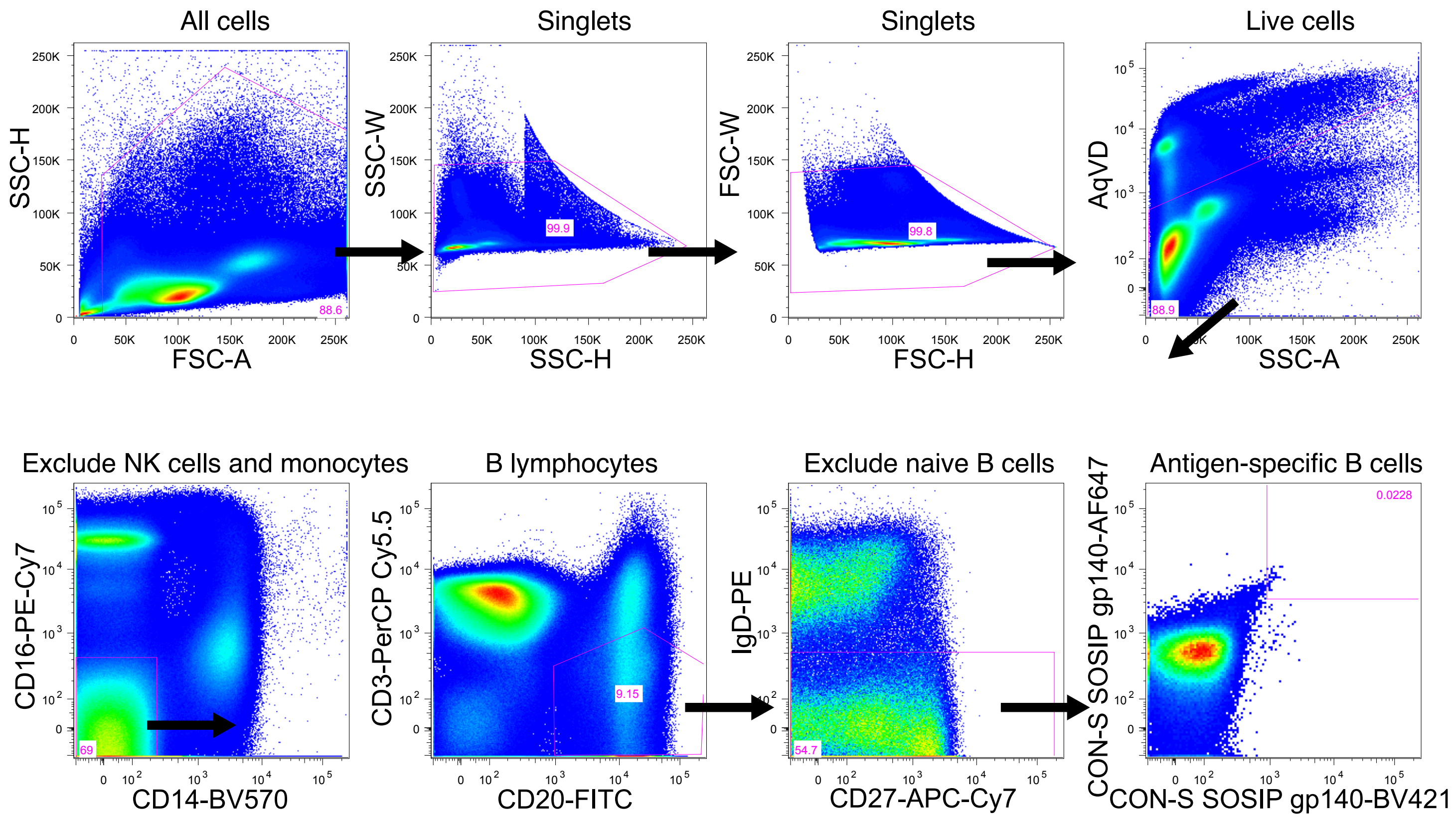

S2 Fig. Fluorescence-activated single-cell sorting of envelope-specific B cells. The gating strategy used to identify M172 week 192 single B cells specific for HIV-1 envelope. B cells from each macaque were sorted with the same strategy shown here. The antigen-specific FACS plot for each macaque is shown in Figure 1.
