# Supplementary material for: Structural and genetic convergence of HIV-1 neutralizing antibodies in vaccinated non-human primates
Source: PLoS Pathog. 2021 Jun 4;17(6):e1009624. doi: 10.1371/journal.ppat.1009624 (PMC8216552; doi:10.1371/journal.ppat.1009624)
Supplement: S4 Fig — (PDF) [file ppat.1009624.s005.pdf]

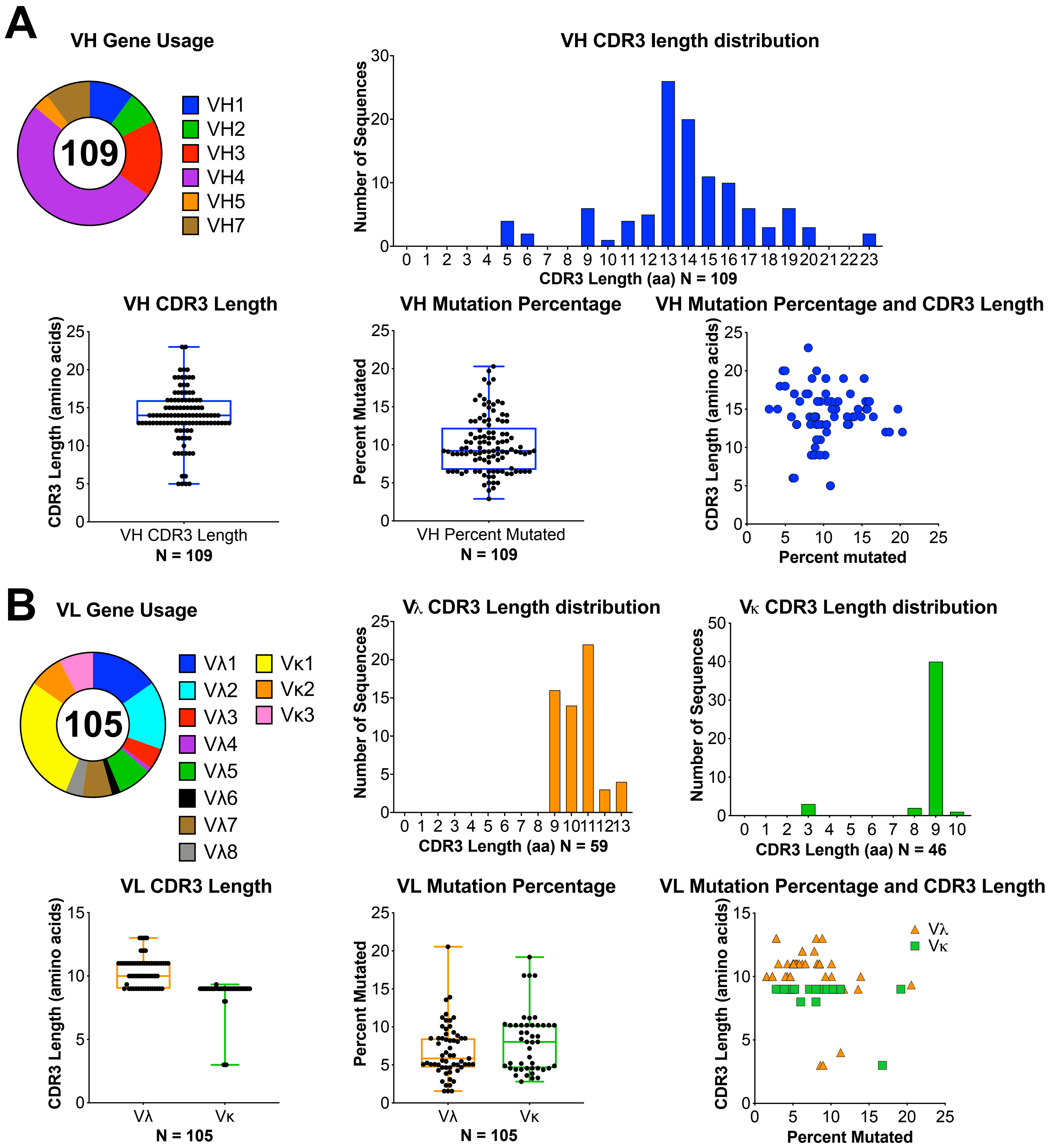

S4 Fig. Immunogenetic analysis of monoclonal antibody sequences isolated from macaque L999. DH840 was among the antibodies isolated from L999. Variable gene segment usage, CDR3 lengths, and mutation percentages are shown for the (**A**) heavy (blue), (**B**) lambda (orange), and (**B**) kappa (green) chains. Genes could not be inferred for 3 lambda chains and 1 kappa chain due to low sequence quality.
