# Supplementary material for: Structural and genetic convergence of HIV-1 neutralizing antibodies in vaccinated non-human primates
Source: PLoS Pathog. 2021 Jun 4;17(6):e1009624. doi: 10.1371/journal.ppat.1009624 (PMC8216552; doi:10.1371/journal.ppat.1009624)
Supplement: S5 Fig — (PDF) [file ppat.1009624.s006.pdf]

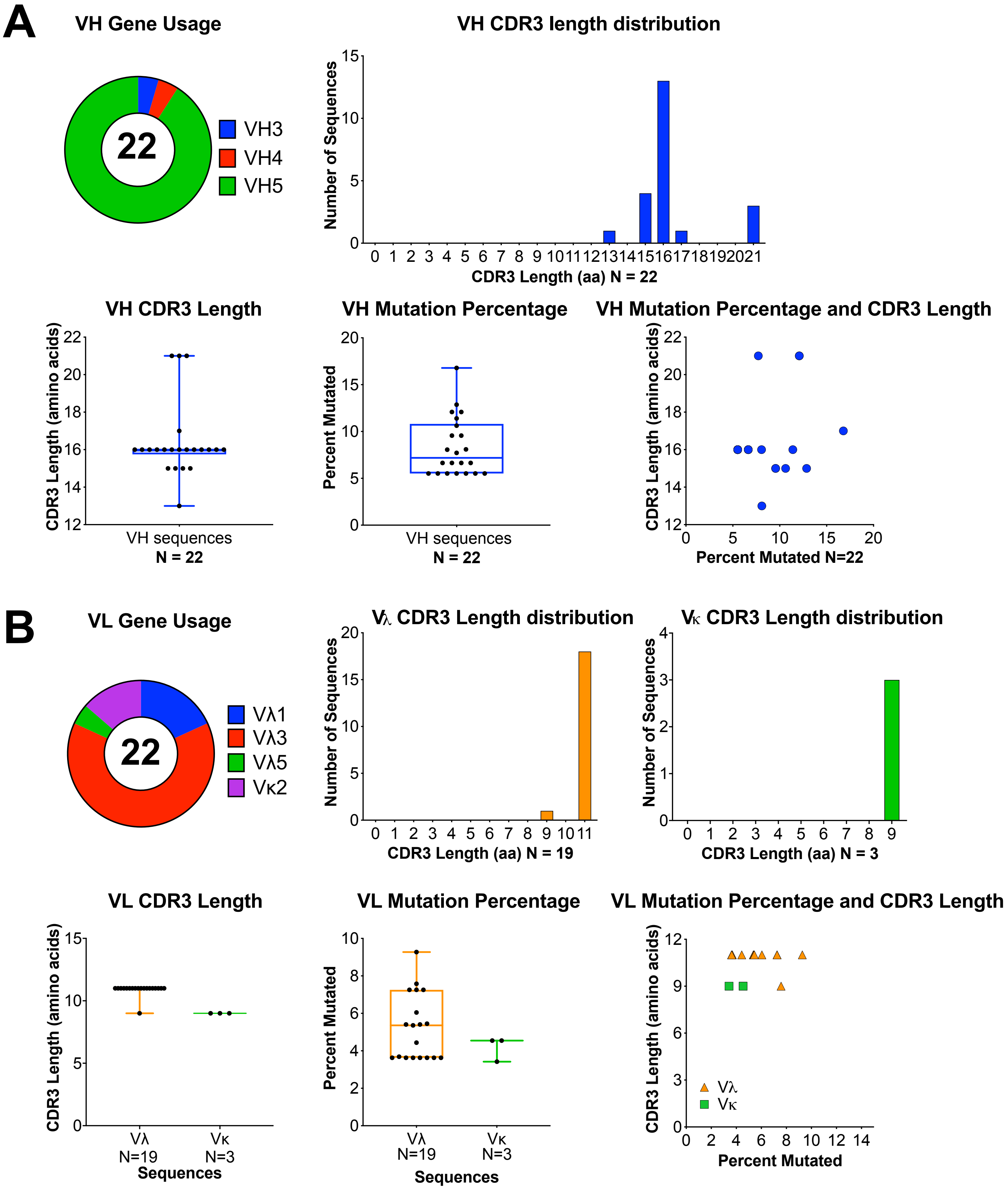

S5 Fig. Immunogenetic analysis of monoclonal antibody sequences isolated from macaque M172 post vaccination. DH842 was among the antibodies isolated from M172. Variable gene segment usage, CDR3 lengths, and mutation percentages are shown for the (**A**) heavy (blue), (**B**) lambda (orange), and (**B**) kappa (green) chains.
