# Supplementary material for: Structural and genetic convergence of HIV-1 neutralizing antibodies in vaccinated non-human primates
Source: PLoS Pathog. 2021 Jun 4;17(6):e1009624. doi: 10.1371/journal.ppat.1009624 (PMC8216552; doi:10.1371/journal.ppat.1009624)
Supplement: S6 Fig — (PDF) [file ppat.1009624.s007.pdf]

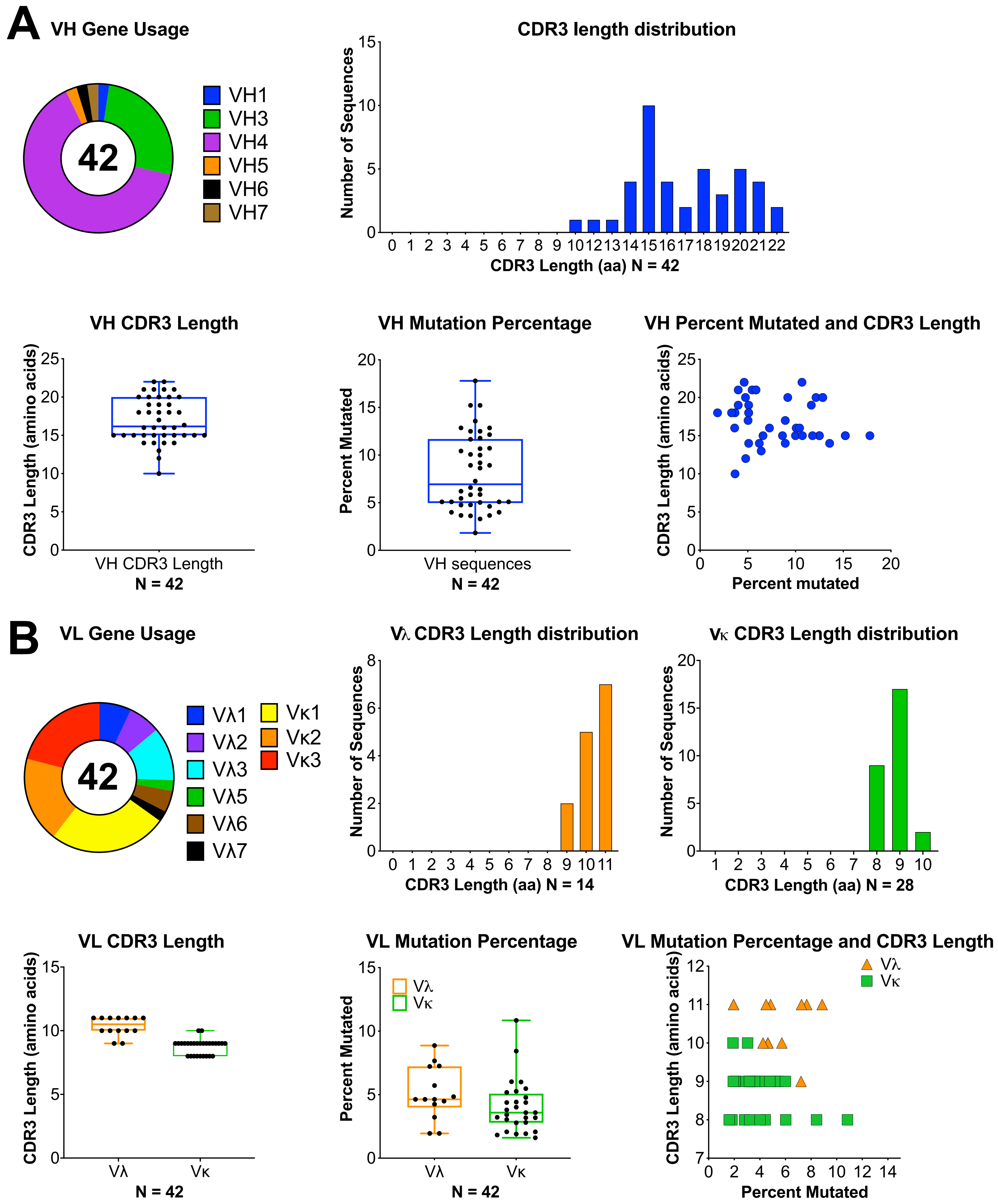

S6 Fig. Immunogenetic analysis of monoclonal antibody sequences isolated from macaque 80-12 post vaccination. DH845 and DH846 lineage antibodies were among the antibodies isolated from 80-12. Variable gene segment usage, CDR3 lengths, and mutation percentages are shown for the (**A**) heavy (blue), (**B**) lambda (orange), and (**B**) kappa (green) chains.
