# Supplementary material for: Structural and genetic convergence of HIV-1 neutralizing antibodies in vaccinated non-human primates
Source: PLoS Pathog. 2021 Jun 4;17(6):e1009624. doi: 10.1371/journal.ppat.1009624 (PMC8216552; doi:10.1371/journal.ppat.1009624)
Supplement: S7 Fig — (PDF) [file ppat.1009624.s008.pdf]

A

Immunogenetics determined by IMGT rhesus macaque library								
Vaccine	Macaque	Antibody	Macaque VH	Macaque JH	HCDR3 length (aa)	Macaque VL	Macaque JL	HCDR3 length (aa)
DNA/Ad5/Protein	L999	DH840.1	IGHV4-2	IGHJ4	15	IGKV3S6	IGKJ4	8
DNA/Ad5/Protein	M172	DH842	IGHV4-2	IGHJ5	17	IGLV5-11	IGLJ1	9
NYVAC-gp120	80-12	DH845.1	IGHV4-2	IGHJ1	20	IGKV1S10	IGKJ2	9
NYVAC-gp120	80-12	DH846.1	IGHV4-2	IGHJ4	15	IGKV3S6	IGKJ1	8
Immunogenetics determined by Clonanalyst rhesus macaque library v2018								
Vaccine	Macaque	Antibody	Macaque VH	Macaque JH	HCDR3 length (aa)	Macaque VL	Macaque JL	HCDR3 length (aa)
DNA/Ad5/Protein	L999	DH840.1	IGHV4-n	IGHJ4	15	IGKV3-d	IGKJ4-1	8
DNA/Ad5/Protein	M172	DH842	IGHV4-n	IGHJ5-1	17	IGLV5-e	IGLJ1	9
NYVAC-gp120	80-12	DH845.1	IGHV4-n	IGHJ1	20	IGKV1-Ab	IGKJ2-1	9
NYVAC-gp120	80-12	DH846.1	IGHV4-n or e	IGHJ4	15	IGKV3-d	IGKJ1-1	8
Immunogenetics determined by Clonanalyst rhesus macaque library v2017								
Vaccine	Macaque	Antibody	Macaque VH	Macaque JH	HCDR3 length (aa)	Macaque VL	Macaque JL	HCDR3 length (aa)
DNA/Ad5/Protein	L999	DH840.1	IGHV4-p	IGHJ4	15	IGKV3-d	IGKJ4-1	8
DNA/Ad5/Protein	M172	DH842	IGHV4-p	IGHJ5-1	17	IGLV5-e	IGLJ1	9
NYVAC-gp120	80-12	DH845.1	IGHV4-p	IGHJ1	20	IGKV1-Ab	IGKJ2-1	9
NYVAC-gp120	80-12	DH846.1	IGHV4-p	IGHJ4	15	IGKV3-d	IGKJ1-1	8

B

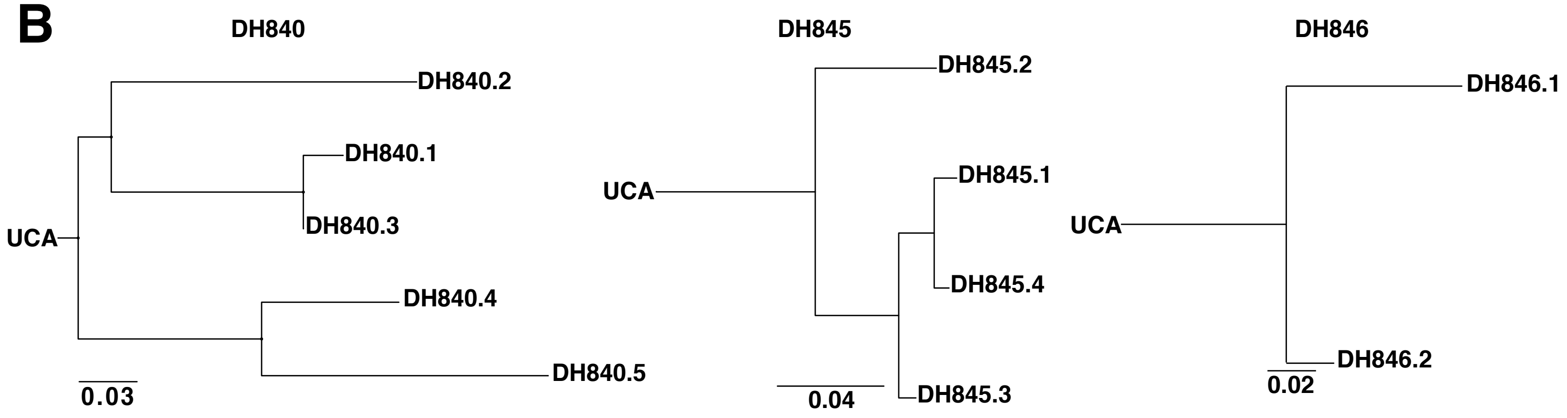

C

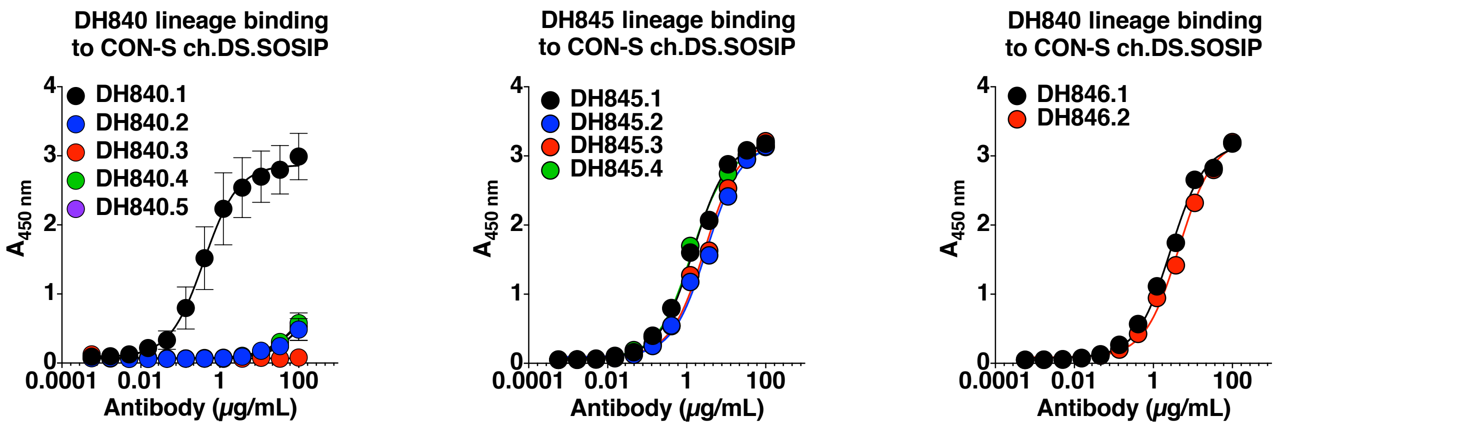

S7 Fig. Clonal analysis of rhesus macaque CON-S neutralizing antibodies. (A) Inference of CON-S neutralizing antibody immunogenetics using different reference gene libraries. Rhesus macaque germline gene usage, and CDR3 lengths were inferred using the rhesus library from IMGT V-QUEST and Cloanalyst with two different versions of a rhesus macaque library. (B) Phylogenetic trees of DH840, DH845, and DH848 lineages. DH842 was the only antibody isolated from its lineage. DH840.1 was isolated as a natural heavy chain paired with a light chain from a single B cell. DH840.2-DH840.5 are heavy chain sequences isolated from VH next-generation sequencing. Clonal lineages were inferred with Cloanalyst software and drawn with FigTree. (C) Antibody binding titers to CON-S envelope trimers by members of each antibody lineage. Binding titer was measured by ELISA, and is shown as the area-under-the-log-transformed curve (log AUC). Values represent the mean and standard error of 3-6 independent experiments.
