# Supplementary material for: Structural and genetic convergence of HIV-1 neutralizing antibodies in vaccinated non-human primates
Source: PLoS Pathog. 2021 Jun 4;17(6):e1009624. doi: 10.1371/journal.ppat.1009624 (PMC8216552; doi:10.1371/journal.ppat.1009624)
Supplement: S8 Fig — (PDF) [file ppat.1009624.s009.pdf]

A

```
DH840.H_UCA QVQLQESGPG LVKPSETLSL TCAVSGYSIS SG.YGWSWIR QPPGKGLEWI  50
   DH840.1H ....K..... ..R....... ......T..N .A..A.G.V. L.........
   DH840.2H .......... .......... ..D...G... GDY.Y.N... ..........
   DH840.5H ..H....... ...S..N.T. I.S....PL. ...L...... .A..R.....

DH840.H_UCA GYIGGSSGST NYNPSLKSRV TISKDTSKNQ FSLKLSSVTA ADTAVYYCAR  100
   DH840.1H MTVYT.T.N. YSD....... .......... ...R.....V E.....F...
   DH840.2H .FL.TYR.TA S.S...R... .F.R...... .....T.... ..........
   DH840.5H .....T.HTA ....P..... I.....F... ...T.K...T ....M....A

DH840.H_UCA ADGYSSGWSY FDYWGQGVLV TVSS  124
   DH840.1H ...SD...PH ..N....L.. ....
   DH840.2H E..DDYANR. .......... S...
   DH840.5H .YY.D..R.. ..G..P.... ..F.
```

B

```
DH842.H_UCA QVQLQESGPG VVKPSETLSL TCAVSGGSIS SGYYYWSWIR QPPGKGLEWI  50
    DH842.H QV........ LMQ.A..... .....RD..G ....A.G.F. .V........

DH842.H_UCA GGIYSNSEST NYNPSLKSRV TISKDTSKNQ FSLKLSSVTA ADTAVYYCAR  100
    DH842.H VT..TSTGNI YDESP..... ...R...... .....T...P ......F...

DH842.H_UCA EGCSSTYCSA GWFDVWGPGV LVTVSS  126
    DH842.H ...NTVF... .........L ......
```

C

```
DH845.H_UCA QVQLQESGPG VVKPSETLSL TCAVSGGSIS SGYYYWSWIR QPPGKGLEWI  50
   DH845.1H .V........ L......... ........M. .V.QG.G... ..........
   DH845.2H .L........ L......... .........N .A.HG.G... ..........
   DH845.3H .L........ L......... ........M. .V.QG.G... ..........
   DH845.4H .V........ L......... ........L. .V.QG.G... ..........

DH845.H_UCA GGIYSNSEST NYNPSLKSRV TISKDTSKNQ FSLKLSSVTA ADTAVYYCAR  100
   DH845.1H ATT.TSTGNI Y......G.G .V.R...... ......A... ..........
   DH845.2H VSS.TSTGN. Y.D....... ...R...... .......... ..........
   DH845.3H ATT.TSTGN. Y.D....... .V.R...... ......A... ..........
   DH845.4H ATT.TSTGNI Y........G .V.R...... ...R..A... ..........

DH845.H_UCA VNLGDSGYYT GVSEYFEFWG QGALVTVSS  129
   DH845.1H L......D.. ......D... .........
   DH845.2H .G...DA.N. .......... .........
   DH845.3H .......D.. ......D... .........
   DH845.4H .......D.. ......D... .........
```

D

```
DH846.H_UCA QVQLQESGPG LVKPSETLSL TCAVSGGSIS DYYYWNWIRQ PPGKGLEWIG  50
   DH846.1H .L........ .......... ..D...V... SN.G.G.... ......D..V
   DH846.2H .......... .......... ......S... VN.G.G.... .........V

DH846.H_UCA NIYGNSASTY YNPSLKSRVT ISKDTSKNQF FLKLSSVTAA DTAVYYCARK  100
   DH846.1H T...GTGN.. .A...EG..A ....A..... S.R.N..... ..........
   DH846.2H T...GTGN.. .D........ ....A.T... S......... ..........

DH846.H_UCA ITYSSGWYYF DYWGQGVLVT VSS  123
   DH846.1H .......... .........A ...
   DH846.2H .......... .......... ...
```

S8 Fig. Amino acid alignments of heavy chain variable regions for each CON-S autologous neutralizing antibody clonal lineage. Amino acids for (A) DH840, (B) DH842, (C) DH845, and (D) DH846 lineages are shown. Periods indicate the presence of identical amino acids at that position. UCAs were inferred with Cloanalyst using the 2017 macaque reference library.
