# Supplementary material for: Structural and genetic convergence of HIV-1 neutralizing antibodies in vaccinated non-human primates
Source: PLoS Pathog. 2021 Jun 4;17(6):e1009624. doi: 10.1371/journal.ppat.1009624 (PMC8216552; doi:10.1371/journal.ppat.1009624)
Supplement: S9 Fig — (PDF) [file ppat.1009624.s010.pdf]

A

```
DH840.K_UCA EIVMTQSPAT LSLSPGERAT LSCRASQSVS SRLAWYQQKP GQAPRLLIYD  50
   DH840.1L ..MV...... ..V....... .........R N.I....... ..S.......

DH840.K_UCA ASSRATGIPD RFSGSGSGTE FTLTISSLEP EDVAVYFCQQ ESNWLTFGGG  100
   DH840.1L ..I..P.... .L........ .....N.... S........L .A........

DH840.K_UCA TKVEIK.... .....  115
   DH840.1L .......... .....
```

B

```
DH842.L_UCA QPVLTQPTSL SASPGASARL TCTLRSGISV GSYRIFWYQQ KPGSPPRYLL  50
    DH842.L .A......A. .......... .......... AD........ R.........

DH842.L_UCA NYHTDSDKHQ GSGVPSRFSG SKDASANAGI LLISGLQSED EADYYCMIWH  100
    DH842.L ..R....H.. .......... ...D...T.F .V........ ....H...R.

DH842.L_UCA NNAYIFGAGT RLTVL  115
    DH842.L .T.....G.. ...L.
```

C

```
DH845.K_UCA DIQMTQSPSA LSASVGDRVT ISCRASQNIY SGLAWYQQKP GKAPKLLIYA  50
   DH845.1L A..L...... .......... .......... .N........ ........FG
   DH845.2L A..L...... .......... .......... .S........ .....V....
   DH845.3L A..L...... .......... .......... .N........ ........FG
   DH845.4L A..L...... .......... .......... .N........ ........FG

DH845.K_UCA ASRLQSGIPS RFSGSGSGTD FTLTISSLQP EDSAAYYCQQ VYSNFYSFGQ  100
   DH845.1L .......... .......... .......... ....T..... ..........
   DH845.2L .......... .......... .......... .......... ..........
   DH845.3L .......... .......... .......... ....T..... ..........
   DH845.4L .......... .......... .......... ....T..... ..........

DH845.K_UCA GTKVEIK... .....  115
   DH845.1L .......... .....
   DH845.2L .......... .....
   DH845.3L .......... .....
   DH845.4L .......... .....
```

D

```
DH846.K_UCA EIVMTQSPAT LSLSPGERAT LSCRASQSVS SRLAWYQQKP GQAPRLLIYD  50
   DH846.1L .......... .......... .......... .......... ..........
   DH846.2L .......... ...F...... .......... T......... ..........

DH846.K_UCA ASSRATGIPD RFSGSGSGTE FTLTISSLEP EDVAVYFCQQ ESNWSTFGQG  100
   DH846.1L ..N....... .......... ......G... .........E DH........
   DH846.2L ..I....... .......... .......... ..F....... DN........

DH846.K_UCA TKVEIK.... .....  115
   DH846.1L .......... .....
   DH846.2L .......... .....
```

S9 Fig. Amino acid alignments of light chain variable regions for each CON-S autologous neutralizing antibody clonal lineages. Amino acids for (A) DH840, (B) DH842, (C) DH845, and (D) DH846 lineages are shown. Periods indicate the presence of identical amino acids at that position. UCAs were inferred with Cloanalyst using the 2017 macaque reference library.
