# Supplementary material for: Structural and genetic convergence of HIV-1 neutralizing antibodies in vaccinated non-human primates
Source: PLoS Pathog. 2021 Jun 4;17(6):e1009624. doi: 10.1371/journal.ppat.1009624 (PMC8216552; doi:10.1371/journal.ppat.1009624)
Supplement: S10 Fig — (PDF) [file ppat.1009624.s011.pdf]

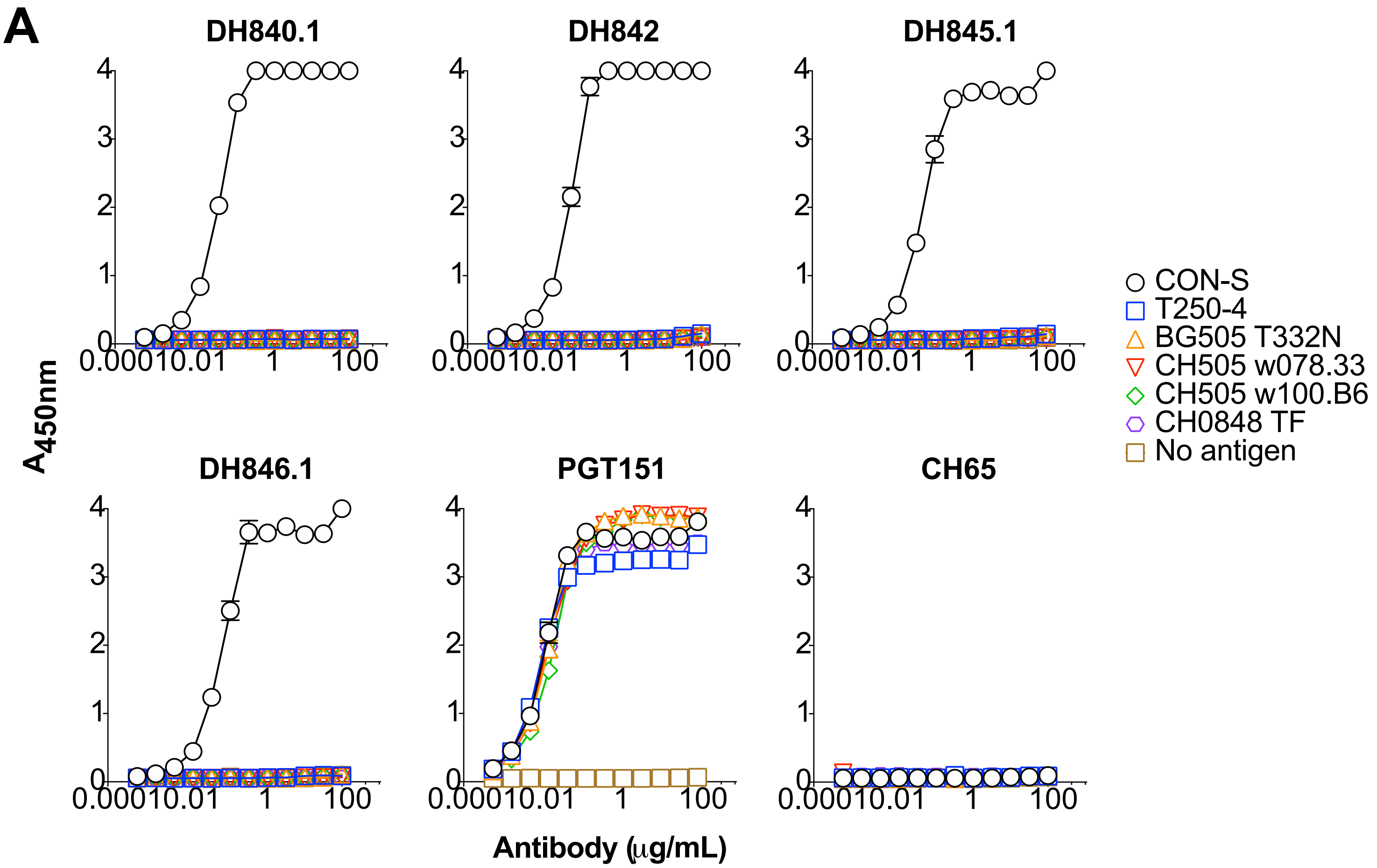

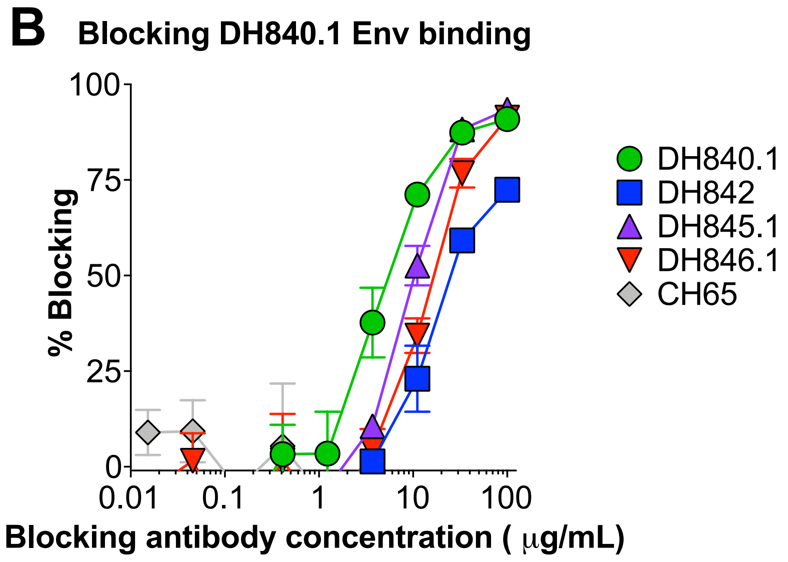

DH840.1 blocking titer (µg/mL)	
**Antibody**	**IC50**
DH840.1	5.7
DH842	24.2
DH845.1	10.6
DH846.1	15.6

S10 Fig. CON-S autologous neutralizing antibodies lack Env trimer binding breadth. (A) Monoclonal antibody binding to autologous and heterologous HIV-1 envelope gp140 SOSIP trimers. Biotinylated SOSIP trimers were captured on streptavidin ELISA plates and examined for binding to each antibody listed in the graph title. Symbols and error bars indicate the mean and standard error of three independent experi-ments. **(B)** CON-S neutralizing antibodies compete with each other for binding to HIV-1 CON-S SOSIP gp140 envelope. The binding of biotinylated DH840.1 was competed with unlabeled CON-S neutralizing antibodies. CH65 was used as a negative control competitor antibody. Mean values and standard error of three indepen-dent experiments are shown. Antibody concentrations that inhibit 50% of DH840.1 binding (IC50) are shown in the table (right side).
