# Supplementary material for: Structural and genetic convergence of HIV-1 neutralizing antibodies in vaccinated non-human primates
Source: PLoS Pathog. 2021 Jun 4;17(6):e1009624. doi: 10.1371/journal.ppat.1009624 (PMC8216552; doi:10.1371/journal.ppat.1009624)
Supplement: S11 Fig — (PDF) [file ppat.1009624.s012.pdf]

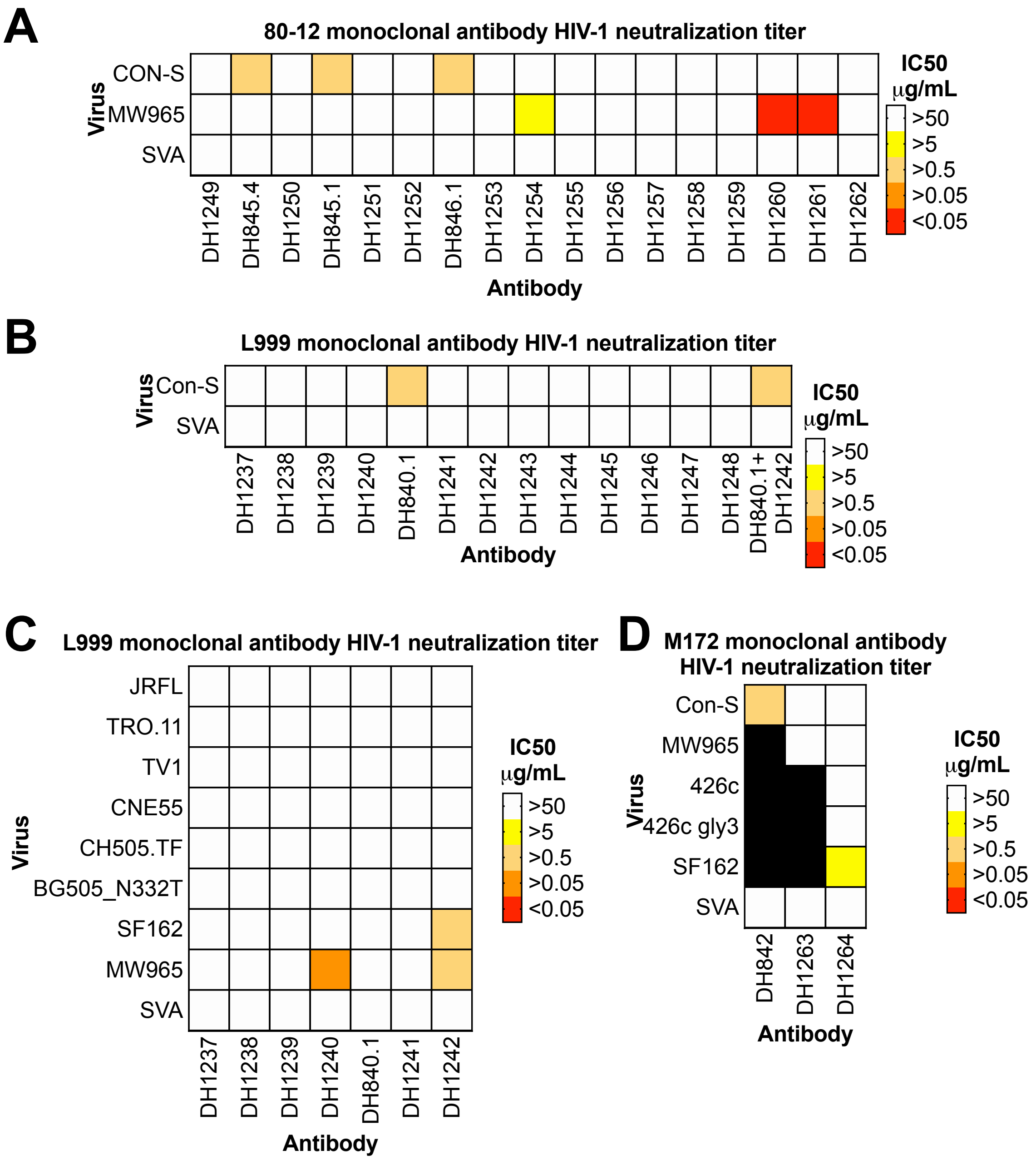

S11 Fig. HIV-1 neutralization titers for rhesus monoclonal antibodies induced by CON-S vaccination. Monoclonal antibodies were isolated from macaques (**A**) 80-12, (**B**, **C**) L999 and (**D**) M172. Each antibody was expressed and tested for neutralization activity against CON-S and other HIV-1 isolates. Neutralization titers are shown as the concentration of antibody that inhibits 50% of virus replication. Titers are color coded based on potency as indicated in the keys. Black squares show viruses not tested for a particular antibody.
