# Supplementary material for: Structural and genetic convergence of HIV-1 neutralizing antibodies in vaccinated non-human primates
Source: PLoS Pathog. 2021 Jun 4;17(6):e1009624. doi: 10.1371/journal.ppat.1009624 (PMC8216552; doi:10.1371/journal.ppat.1009624)
Supplement: S12 Fig — (PDF) [file ppat.1009624.s013.pdf]

A

```
IGHV4-n*03|AR212    CAGGTGCAGCTGCAGGAGTCGGGCCCAGGACTGGTGAAGCCTTCGGAGACCCTGTCCCTCACCTGCGCTGTCTCTGGTTACTCCATCAGCAGTGGTTACT
DH846.1 VH          ...C...............................................................A..........GT............CAA...TG
DH846.2 VH          .............................................................................GAG..........GT.AA...TG

IGHV4-n*03|AR212    ACTGGGGCTGGATCCGTCAGCCCCCAGGGAAGGGACTGGAGTATATTGGGTATATCAGTGGTAGTAGTGGGAGCACCTACTACAACCCCTCCCTCAAGAG
DH846.1 VH          G...............C........T..............C.GG....T.AC....TA...CG...CG..A.A..........GC...T......G..G.
DH846.2 VH          G...............C.................G.......GG....T.AC....TA....G...CG....A..........G................

IGHV4-n*03|AR212    TCGAGTCACCATTTCAAAAGACACGTCCAAGAACCAGTTCTCCCTGAAGCTGAGCTCTGTGACCGCCGCGGACACGGCCGTGTATTACTGTGCGAGA
DH846.1 VH          ......TG..............G.A......................G.....A......A....................A.....T........G
DH846.2 VH          ............C.........G.A....C..................A...............................................G
```

B

```
IGHV4-e*01|AR194    CAGGTGCAGCTGCAGGAGTCGGGCCCAGGACTGGTGAAGCCTTCGGAGACCCTGCCCCTCACCTGCGCTGTCTCTGGTGCCTCCATCAGCAGTAACTA--
DH846.1 VH          ...C..................................................T............A...........T............C..T..TG
DH846.2 VH          ......................................................T......................GAG..........GT...T..TG

IGHV4-e*01|AR194    -CTGGAGCTGGATCCGCCAGCCCCCAGGGAAGGGACTGGAGTGGATTGGGTATATCTATGGTGGTAGTGGGAGCACCAGCTACAACCCCTCCCTCAAGAG
DH846.1 VH          G....G...................T..............C.......T.AC.........C....CG..A.A....TA....GC...T......G..G.
DH846.2 VH          G....G............................G.............T.AC..............CG....A....TA....G................

IGHV4-e*01|AR194    TCGAGTCACCATTTCCAAAGACACGTCCAAGAACCAGTTCTCCCTGAAGCTGAGCTCTGTGACCGCCGCGGACACGGTCGTGTATTACTGTGCGAGA
DH846.1 VH          ......TG.......A......G.A......................G.....A......A................C...A.....T........G
DH846.2 VH          ............C..A......G.A....C..................A............................C..................G
```

C

```
IGHV4-f*03|AR197    CAGGTGCAGCTGCAGGAGTCGGGCCCAGGACTGGTGAAGCCTTCGGAGACCCTGTCCCTCACCTGCGCTGTCTCTGGTGGCTCCATCAGCGATTACTACT
DH846.1 VH          ...C...............................................................A...........T..........AGCA.T..TG
DH846.2 VH          .............................................................................GA............T.A.T..TG

IGHV4-f*03|AR197    ACTGGAACTGGATCCGCCAACCCCCAGGGAAGGGGCTGGAGTGGATTGGGAATATCTATGGTAATAGTGCGAGCACCTACTACAACCCCTCCCTCAAGAG
DH846.1 VH          G....GG............G.....T........A.....C.......T..C.........CGG..CG.GA.A..........GC...T......G..G.
DH846.2 VH          G....GG............G............................T..C..........GG..CG.G..A..........G................

IGHV4-f*03|AR197    TCGAGTCACCATTTCAAAAGACACGTCCAAGAACCAGTTCTTCCTGAAGCTGAGCTCTGTGACCGCCGCGGACACCGCCGTGTATTACTGTGCGAGA
DH846.1 VH          ......TG..............G.A................C.....G.....A......A..............G.....A.....T........G
DH846.2 VH          ............C.........G.A....C...........C......A..........................G....................G
```

D

Reference VH gene segment comparison with DH846.1 and DH846.2				
VH	Mismatches with DH840.1 and 2	VH divergence (%)		Indels
		DH846.1	DH846.2	
IGHV4-n	21	13.7	10	0
IGHV4-e	20	13.1	9.6	3
IGHV4-f	22	14.8	9.3	0

S12 Fig. Comparison of DH846.1 and DH846.2 nucleotide sequence with putative rhesus macaque germline V gene segments. (A-C) Nucleotide alignments for the three rhesus macaque germline V gene segments with highest sequence identity to DH846.1 and DH846.2. HCDRs are bolded. Nucleotides that are the same in DH846.1 and DH846.2, but different in the reference gene segment are highlighted yellow, and the total number of these differences is shown in (D). IGHV4-e, but not the other two reference V gene segments, contained a 3 nucleotide insertion (indels) in HCDR2 compared to DH846.1 and DH846.2 antibodies.
