# Supplementary material for: Structural and genetic convergence of HIV-1 neutralizing antibodies in vaccinated non-human primates
Source: PLoS Pathog. 2021 Jun 4;17(6):e1009624. doi: 10.1371/journal.ppat.1009624 (PMC8216552; doi:10.1371/journal.ppat.1009624)
Supplement: S13 Fig — (PDF) [file ppat.1009624.s014.pdf]

```
           FR1                   CDR1   FR2        CDR2        FR3
DH840.1H QVQLKESGPGLVRPSETLSLTCAVSGTSINSAYAWGWVRLPPGKGLEWIMTVYTSTGNTYSDPSLKSRVT
DH846.1H .L..Q.......K.........D...V..S.N.G...I.Q......D..V.I.GG.....YA...EG..A

           FR3                 CDR3       FR4
DH840.1H ISKDTSKNQFSLRLSSVTVEDTAVYFCARADGSDSGWPHFDNWGQGLLVTVSS  123
DH846.1H ....A.........N...AA.....Y...KITYS...YY..Y....V..A...


            FR1             CDR1        FR2          CDR2          FR3
DH840.1L EIMVTQSPATLSVSPGERATLSCRASQSVRNRIAWYQQKPGQSPRLLIYDASIRAPGIPDRLSGSGSGTEFTLTINSLEP
DH846.1L ..VM........L................SS.L.........A.........N..T.....F.............SG...

          FR3    CDR3    FR4
DH840.1L SDVAVYFCQLEANWLTFGGGTKVEIK
DH846.1L E........EDH..S...Q.......
```

S13 Fig. Amino acid alignment of DH840.1 and DH846.1 variable regions.
Complementarity determining regions (CDR) and framework regions (FR) are designated using the Kabat definitions for the heavy chain (H, top) and light chain (L, bottom) variable regions. Periods indicate identical amino acids.
