# Supplementary material for: Structural and genetic convergence of HIV-1 neutralizing antibodies in vaccinated non-human primates
Source: PLoS Pathog. 2021 Jun 4;17(6):e1009624. doi: 10.1371/journal.ppat.1009624 (PMC8216552; doi:10.1371/journal.ppat.1009624)
Supplement: S14 Fig — (PDF) [file ppat.1009624.s015.pdf]

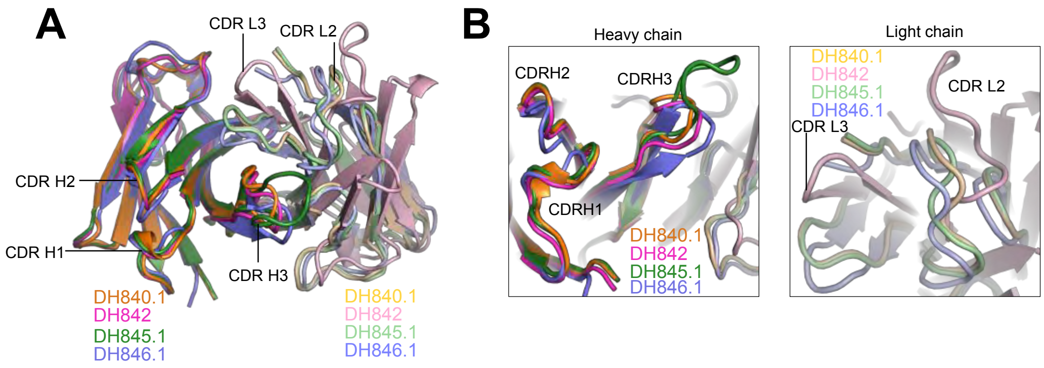

C

```
                                                  CDRH1                          CDRH2                                                   CDRH3
DH840.1_H      QVQLKESGPGLVRPSETLSLTCAVSGTSINSAYAWGWVRLPPGKGLEWIMTVYTSTGNTYSDPSLKSRVTISKDTSKNQFSLRLSSVTVEDTAVYFCARADGSDS-----GWPHFDNW
DH842_H        QVQLQESGPGLMQPAETLSLTCAVSRDSIGSGYAWGWFRQVPGKGLEWIVTIYTSTGNIYDESPLKSRVTISRDTSKNQFSLKLTSVTPADTAVYFCAREGCNTV---FCSAGWFDVW
DH845.1_H      QVQLQESGPGLVKPSETLSLTCAVSGGSMSSVQGWGWIRQPPGKGLEWIATTYTSTGNIYYNPSLKGRGTVSRDTSKNQFSLKLSAVTAADTAVYYCARLNLGDSGDYTGVSEYFDFW
DH846.1_H      QLQLQESGPGLVKPSETLSLTCDVSGVSISSNYGWGWIRQPPGKGLDWIVTIYGGTGNTYYAPSLEGRVAISKDASKNQFSLRLNSVTAADTAVYYCARKITYSS-----GWYYFDYW
               *:**:******::*:******* **   *:.*  .***.*  *****:** * * .*** *    *:.* ::*:*:*******:*.:**  *****:***                ** *

                                             CDRL1                             CDRL2                                       CDRL3
DH840.1_KC     EIMVTQSPATLSVSPGERATLSCRASQSV---RNRIAWYQQKPGQSPRLLIYDA----SIRAPGIPDRLSGSGSGT--EFTLTINSLEPSDVAVYFCQL-EANWLTF
DH842_LC       -QAVLTQPTALSASPGASARLTCTLRSGISVADYRIFWYQQRPGSPPRYLLNYRTDSDHHQGSGVPSRFSGSKDDSANTGFLVISGLQSEDEADYHCMIRHNTAYIF
DH845.1_KC     AIQLTQSPSALSASVGDRVTISCRASQNI---YSNLAWYQQKPGKAPKLLIFGA----SRLQSGIPSRFSGSGSGT--DFTLTISSLQPEDSATYYCQQVYSNFYSF
DH846.1_KC     EIVMTQSPATLSLSPGERATLSCRASQSV---SSRLAWYQQKPGQAPRLLIYDA----SNRATGIPDRFSGSGSGT--EFTLTISGLEPEDVAVYFCQE-DHNWSTF
                  :  .*::** * *  . ::*   ..:     .: ****:**. *: *:           *:*.*:*** ..:     *.*..*: .* * *.*     .   *
```

S14 Fig. Model of DH842 and DH845 Fab molecules. (A) Overlay of DH840.1, and DH846 crystal structures with models of DH842, and DH845 Fab molecules shown in ribbon representation. (B) Zoom-in of the heavy chain and light chain. (C) Sequence alignment of the DH840.1, DH842, DH845, and DH846 heavy and light chain sequences. CDR regions are shaded in gray.
