# Supplementary material for: Structural and genetic convergence of HIV-1 neutralizing antibodies in vaccinated non-human primates
Source: PLoS Pathog. 2021 Jun 4;17(6):e1009624. doi: 10.1371/journal.ppat.1009624 (PMC8216552; doi:10.1371/journal.ppat.1009624)
Supplement: S15 Fig — (PDF) [file ppat.1009624.s016.pdf]

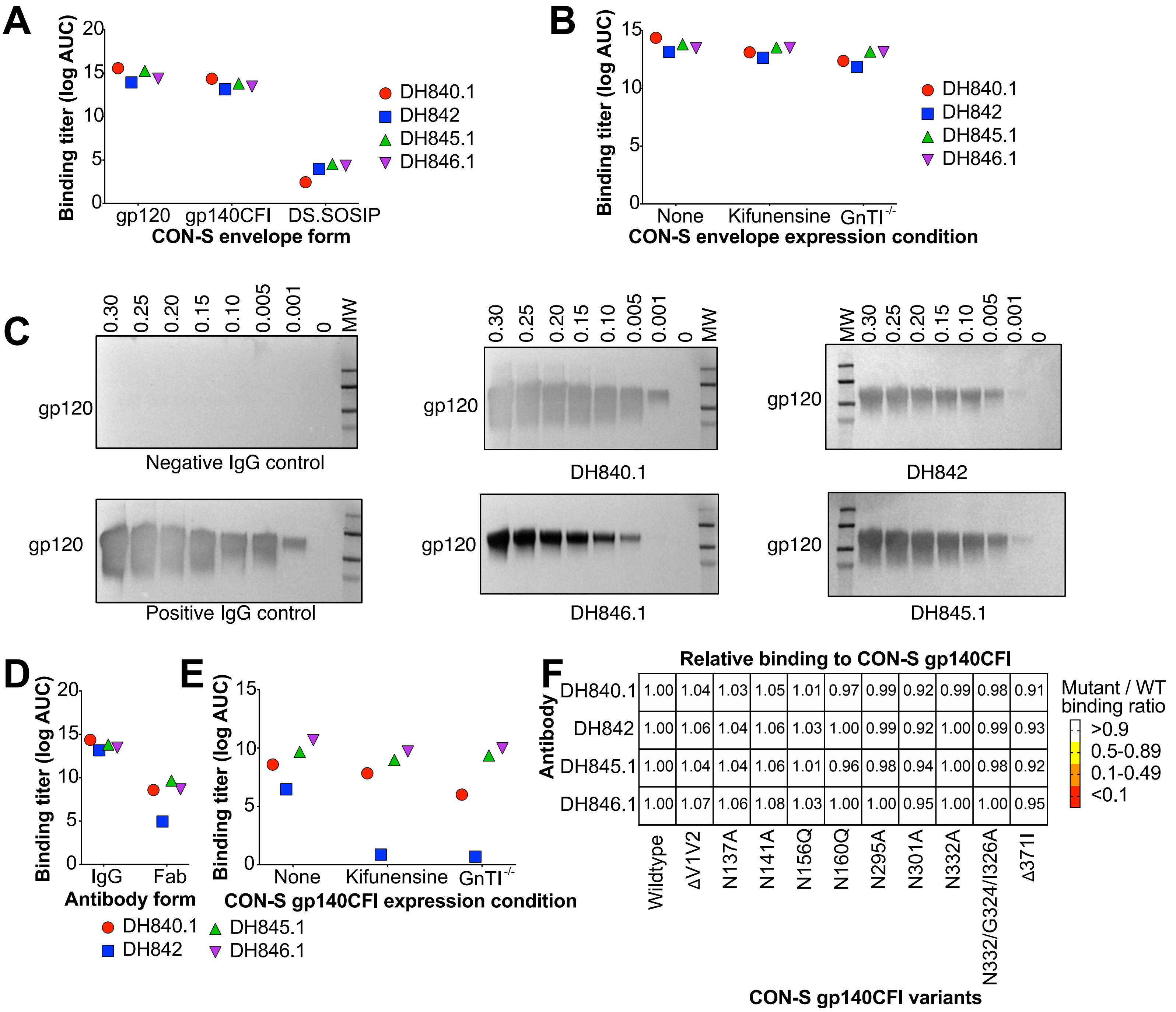

S15 Fig. Antibody recognition of different CON-S envelope subunits and glycoforms. (**A**,**B**) Recombinant IgG binding titers to CON-S envelope as measuserd by ELISA. Glycoform variants shown in (B) are CON-S gp140CFI. Titers are shown as area under the log-trans-formed curve (log AUC). (**C**) Recombinant IgG binding to gp120 by western blot under reduced conditions. Anti-flu IgG and V3-specific antibody 19B were used as negative and postive controls respectively. (**D**) Comparison of IgG and Fab binding for each antiobdy to CON-S gp140CFI. (**E**) Comparison of Fab binding to different glycofroms of CON-S gp140CFI. Glycosylation of the gp140 was enriched for $Man_9GlcNAc_2$ or $Man_5GlcNAc_2$ glycosylation by expression in GnTI$^{-/-}$ cells or kifunensine treatment. (**F**) Relative binding to CON-S gp140CFI with mutations in known bnAb sites. Binding is normalized to wild-type CON-S gp140CFI binding titer. Mean values of two independent experiments are shown in each panel.
