# Supplementary material for: Structural and genetic convergence of HIV-1 neutralizing antibodies in vaccinated non-human primates
Source: PLoS Pathog. 2021 Jun 4;17(6):e1009624. doi: 10.1371/journal.ppat.1009624 (PMC8216552; doi:10.1371/journal.ppat.1009624)
Supplement: S16 Fig — (PDF) [file ppat.1009624.s017.pdf]

A

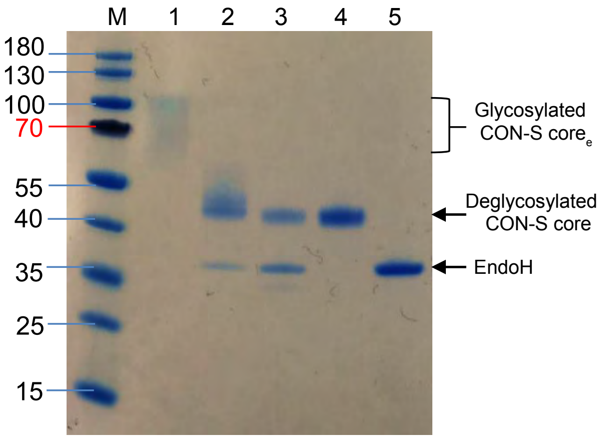

B

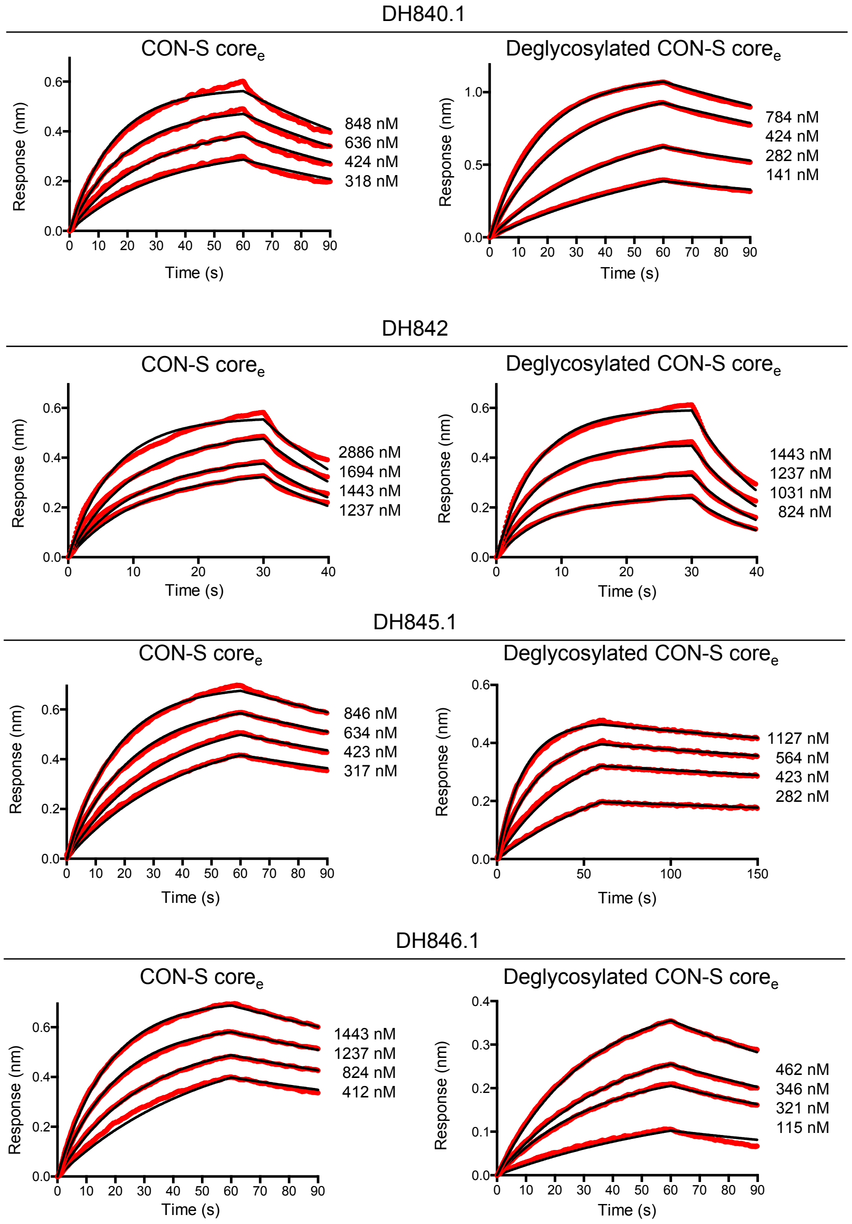

C

CON-S-Core-e	K_D (M)	K_D Error	k_{on}(1/Ms)	k_{dis}(1/s)
DH840.1	1.64E-07	3.68E-09	6.54E+04	1.07E-02
DH842	1.35E-06	6.46E-08	3.38E+04	4.55E-02
DH845.1	6.95E-08	7.54E-10	6.50E+04	4.51E-03
DH846.1	1.20E-07	2.05E-09	3.69E+04	4.42E-03

D

degly CON-S-Core-e	K_D (M)	K_D Error	k_{on}(1/Ms)	k_{dis}(1/s)
DH840.1	4.57E-08	5.91E-10	1.19E+05	5.43E-03
DH842	1.38E-06	5.40E-08	5.79E+04	7.97E-02
DH845.1	1.51E-08	1.64E-10	1.81E+05	2.73E-03
DH846.1	1.46E-07	3.16E-09	5.30E+04	7.71E-03

S16 Fig. Binding kinetics of CON-S nAbs to glycosylated and deglycosylated CON-S core$_e$ measured by Biolayer Interferometry. (**A**) SDS-PAGE of purified CON-S core$_e$ gp120 prior to and following EndoH-based deglycosylation. M: Protein ladder; 1: CON-S-core$_e$; 2: CON-S-core$_e$ + EndoH; 3: CON-S-core$_e$ + EndoH following ConA-resin purification; 4: Deglycosylated CON-S-core$_e$ following size-exclusion purification; 5: EndoH following size-exclusion purification. (**B**) Affinity measurements of CON-S nAbs to glycosylated and deglycosylated CON-S core$_e$ gp120 as measured by Biolayer Interferometry. (**C,D**) CON-S autologous neutralizing antibodies binding kinetics to glycosylated and deglycosylated CON-S Core$_e$. Values were calculated from the curves in B.
