# Supplementary material for: Structural and genetic convergence of HIV-1 neutralizing antibodies in vaccinated non-human primates
Source: PLoS Pathog. 2021 Jun 4;17(6):e1009624. doi: 10.1371/journal.ppat.1009624 (PMC8216552; doi:10.1371/journal.ppat.1009624)
Supplement: S17 Fig — (PDF) [file ppat.1009624.s018.pdf]

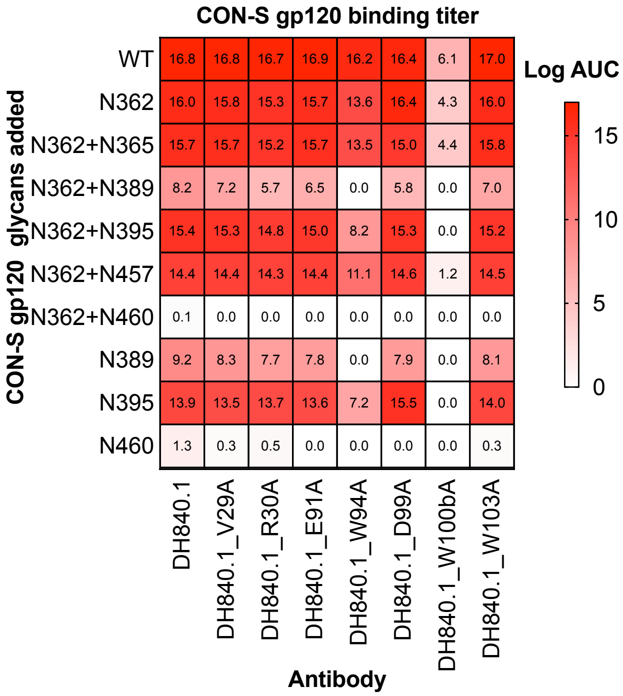

S17 Fig. The structure and function of the DH840.1 paratope demonstrates $W94_{CDR3}$ and $W100b_{CDR3}$ are critical for DH840.1 binding to hyperglycosylated CON-S envelope gp120. Hyperglycosylated CON-S gp120 binding titers (log AUC) of DH840.1 mutated at exposed amino acids. Each row shows the binding titer for wildtype and CON-S gp120 with glycans added at the designated positions. Tryptophans at position 94 and 100b in the light and heavy chain third complementarity determining region (CDR3) respectively were required for accommodating N389, N395, and N457 glycans. Mean values of two independent experiments are shown.
