# Supplementary material for: Structural and genetic convergence of HIV-1 neutralizing antibodies in vaccinated non-human primates
Source: PLoS Pathog. 2021 Jun 4;17(6):e1009624. doi: 10.1371/journal.ppat.1009624 (PMC8216552; doi:10.1371/journal.ppat.1009624)
Supplement: S18 Fig — (PDF) [file ppat.1009624.s019.pdf]

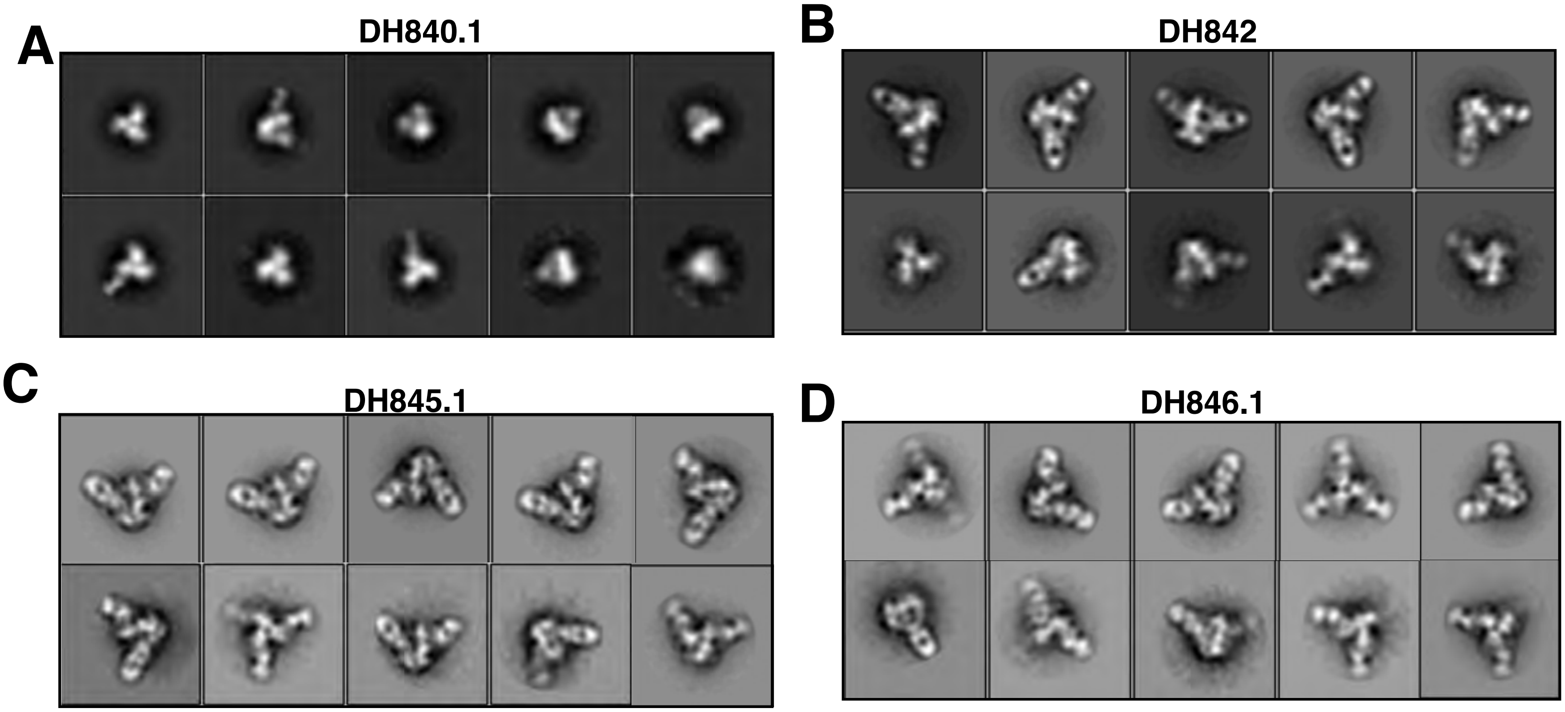


**S18 Fig. Negative-stain electron microscopy single-particle 2D class averages of HIV-1 CON-S DS.SOSIP.664 trimer bound to (A) DH840.1, (B) DH842, (C) DH845.1, and (D) DH846.1 Fabs.**
