# Supplementary material for: Structural and genetic convergence of HIV-1 neutralizing antibodies in vaccinated non-human primates
Source: PLoS Pathog. 2021 Jun 4;17(6):e1009624. doi: 10.1371/journal.ppat.1009624 (PMC8216552; doi:10.1371/journal.ppat.1009624)
Supplement: S19 Fig — (PDF) [file ppat.1009624.s020.pdf]

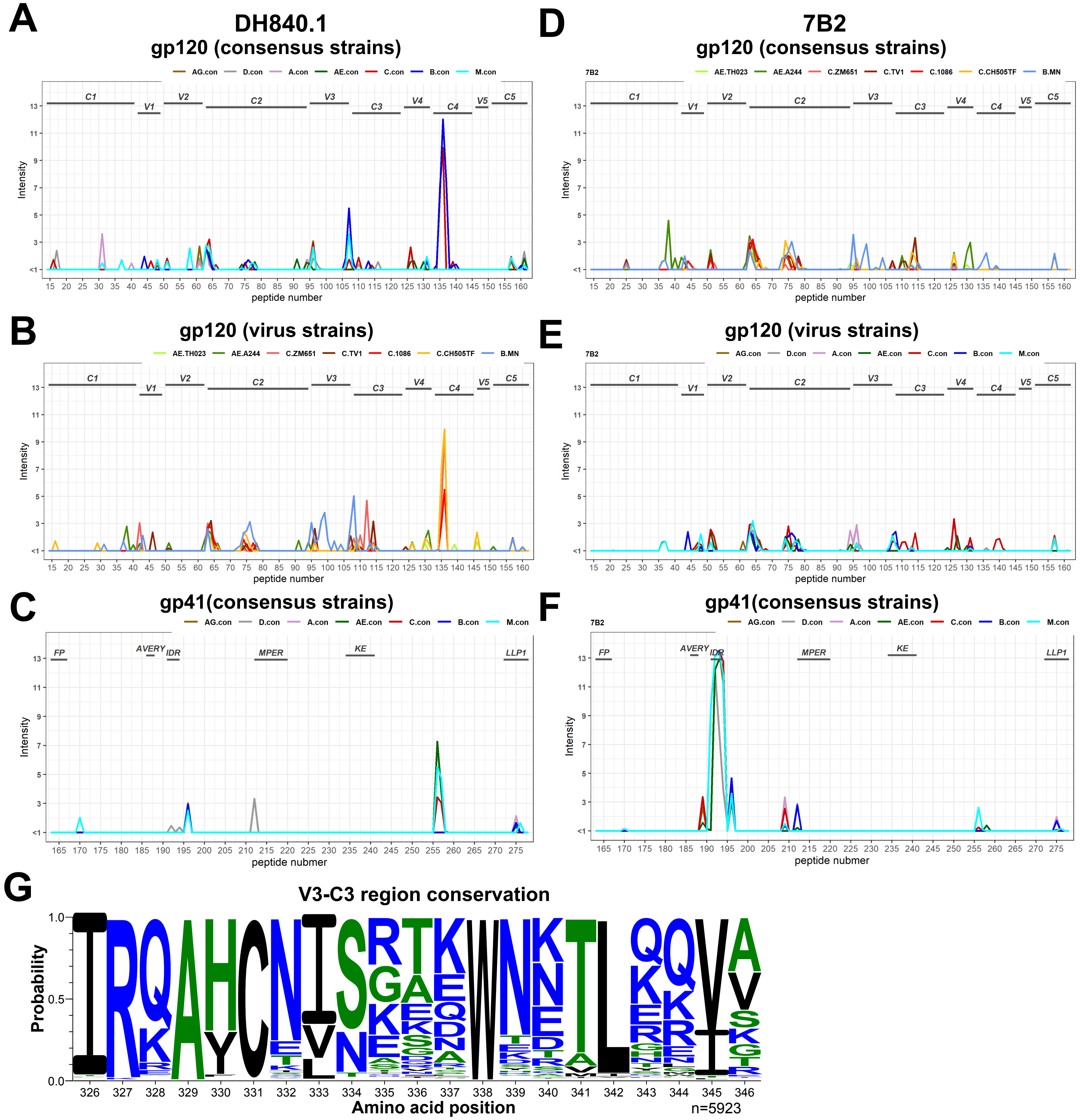

S19 Fig. DH840.1 reactivity with HIV-1 envelope gp120 and gp41 peptides. (**A-C**) Vac-cine-elicited rhesus antibody DH840.1 reactivity with HIV-1 envelope peptides derived from consensus and wild-type virus strains was determined by peptide microarray. Each strain is shown as a different color line. Each peptide is fifteen amino acids long and overlaps with neighboring peptides by three amino acids. M.con refers to a consensus group M envelope that is different from CON-S. While the C4 peptide was bound strongly for many HIV-1 strains the peptide matching the CON-S C4 sequence was not bound. (**D-F**) Human gp41-reactive antibody 7B2 was used as a positive control. Values within an assay are considered positive if greater than 3 times the intensity value of negative control polyclonal immunoglobulin IVIG and greater than the 95th percentile of neg control CH65 immunoglobulin binding to all peptides. Overall, values are shown as positive if they were positive in two independent assays. Values shown are representative of the two independent arrays. (**G**) LOGO plot of amino acid prevalence in the V3-C3 region bound by DH840.1 in the peptide array. Image generated with AnalyzeAlign. This region included conserved amino acids interspersed between highly variable amino acids. Positions 326, 327, 329, 331, 338, and 342 were 95.6-99.8% conserved. The other 15 amino acids ranged in conservation from 27-83%.
