# Supplementary material for: Structural and genetic convergence of HIV-1 neutralizing antibodies in vaccinated non-human primates
Source: PLoS Pathog. 2021 Jun 4;17(6):e1009624. doi: 10.1371/journal.ppat.1009624 (PMC8216552; doi:10.1371/journal.ppat.1009624)
Supplement: S20 Fig — (PDF) [file ppat.1009624.s021.pdf]

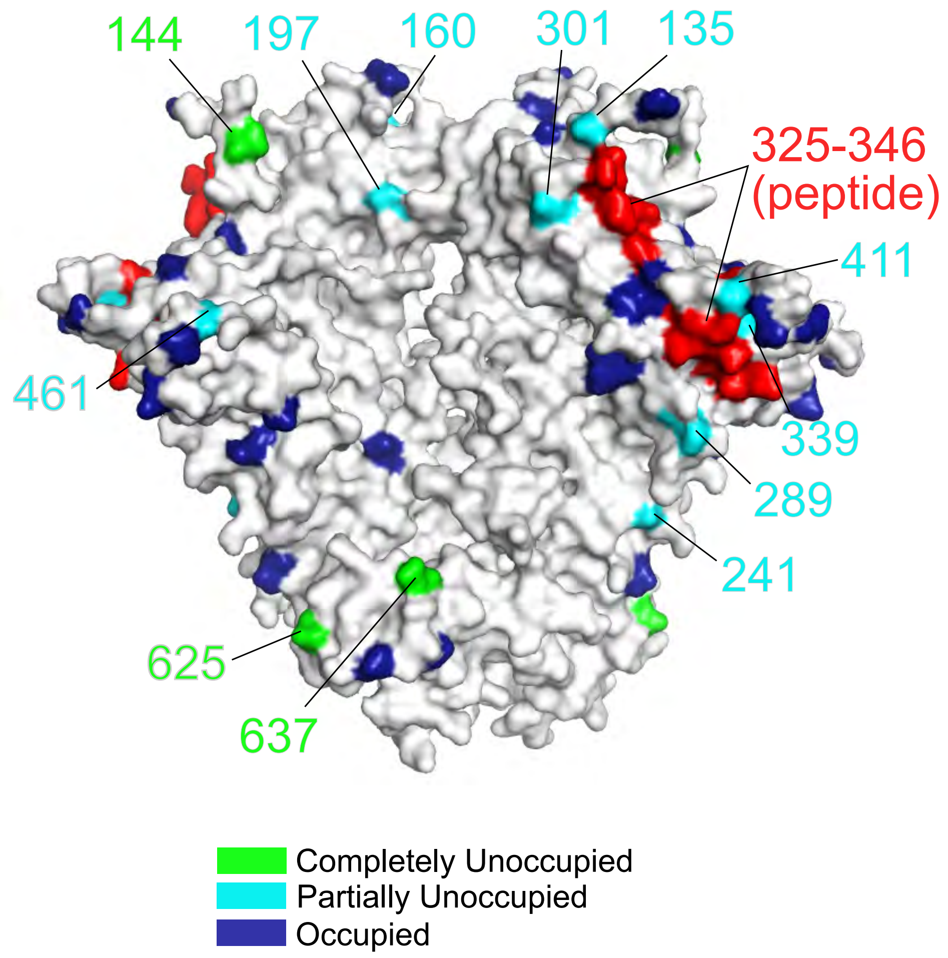

S20 Fig. Complete and partially unoccupied glycosylation sites in CON-S gp140. This figure shows the glycan sites that were found to be completely uoccupied (green) or partially unoccupied (cyan) in CON-S gp140 CFI, using data from Go et al.[53]. Other glycan sites found to be completely occupied are shown in dark blue. The site of DH840.1 contact in V3-C3 (positions 325-346) is shown in red. The crystal structure of X1193 trimer is used (PDB: 5FYJ) with hypervariable loops truncated to match those of CON-S.
