# Supplementary material for: Structural and genetic convergence of HIV-1 neutralizing antibodies in vaccinated non-human primates
Source: PLoS Pathog. 2021 Jun 4;17(6):e1009624. doi: 10.1371/journal.ppat.1009624 (PMC8216552; doi:10.1371/journal.ppat.1009624)
Supplement: S3 Fig — (PDF) [file ppat.1009624.s004.pdf]

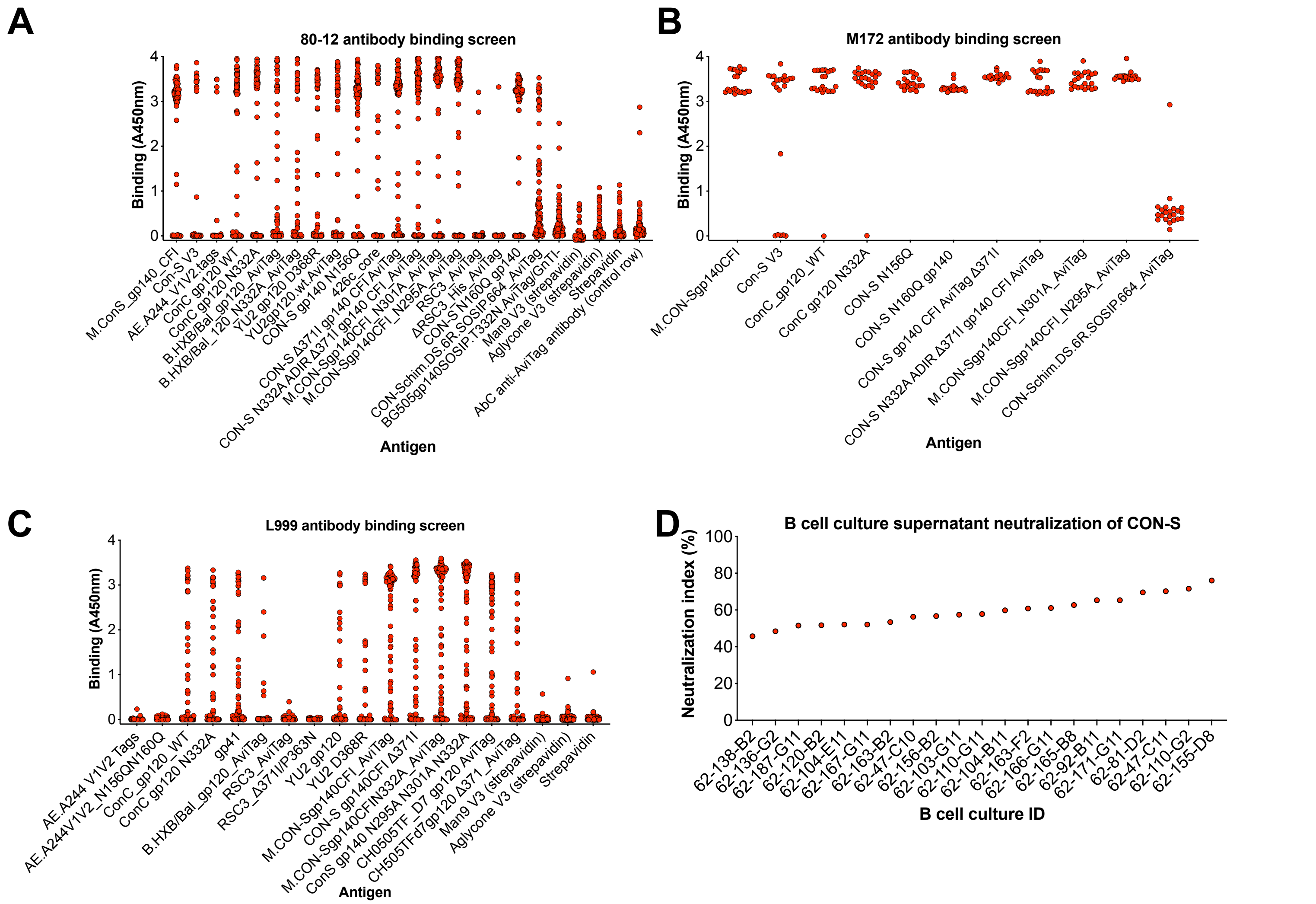

S3 Fig. Small-scale high-throughput characterization of binding reactivity and CON-S neutralization by cell culture supernatants containing monoclonal antibodies from antigen-specific B cells from immunized macaques. Binding reactivities of monoclonal antibodies isolated from macaques (**A**) 80-12, (**B**) M172, or (**C**) L999. (**D**) L999 antigen-specific B cells were cultured in vitro and cell culture supernatants were screened for CON-S neutralization. Shown are the 21 of 11,639 B cell cultures that exhibited CON-S neutralization activity. Antibodies were isolated from these cultures and gave rise to DH840.1.
